# Supplementary material for: The sensitivity of TANDEM – A new measure of trauma competence
Source: PLoS One. 2026 Jan 28;21(1):e0339858. doi: 10.1371/journal.pone.0339858 (PMC12851466; doi:10.1371/journal.pone.0339858)
Supplement: S1 Table — (DOCX) [file pone.0339858.s001.docx]

Supplementary Table 1: Measurement invariance results

|  |  | Readiness ^a^ | | | | | |  |  | Agency ^b^ | | | | | |  |  | Reflexivity ^c^ | | | | | |  |
| --- | --- | --- | --- | --- | --- | --- | --- | --- | --- | --- | --- | --- | --- | --- | --- | --- | --- | --- | --- | --- | --- | --- | --- | --- |
|  |  | χ^2^ | df | P | CFI | TLI | RMSEA | |  | χ^2^ | df | P | CFI | TLI | RMSEA | |  | χ^2^ | df | P | CFI | TLI | RMSEA | |
| Configural |  | 1.47 | 5 | .917 | 1.00 | 1.00 | .000 |  |  | 60.045 | 29 | .001 | 0.96 | 0.94 | 0.054 |  |  | 64.717 | 29 | .000 | 0.95 | 0.92 | 0.058 |  |
| Weak (metric) |  | 5.76 | 7 | .568 | 1.00 | 1.00 | .000 |  |  | 63.649 | 33 | .001 | 0.96 | 0.95 | 0.050 |  |  | 67.854 | 33 | .000 | 0.95 | 0.94 | 0.054 |  |
| ∆ |  | 4.29 | 2 | .117 | 0.00 | 0.00 | .000 |  |  | 3.604 | 4 | .462 | 0.00 | 0.01 | -0.004 |  |  | 3.137 | 4 | .535 | 0.00 | 0.01 | -0.004 |  |
|  |  |  |  |  |  |  |  |  |  |  |  |  |  |  |  |  |  |  |  |  |  |  |  |  |
| Strong (scalar) |  | 13.73 | 9 | .132 | 0.99 | 0.98 | .038 |  |  | 102.211 | 38 | .000 | 0.92 | 0.90 | 0.068 |  |  | 105.765 | 38 | .000 | 0.91 | 0.89 | 0.070 |  |
| ∆ |  | 7.97 | 2 | .019 | -0.01 | -0.02 | .038 |  |  | 38.562 | 5 | .000 | -0.04 | -0.04 | 0.018 |  |  | 37.911 | 5 | .000 | -0.04 | -0.05 | 0.016 |  |
|  |  |  |  |  |  |  |  |  |  |  |  |  |  |  |  |  |  |  |  |  |  |  |  |  |
| Strict |  | 26.74 | 13 | .014 | 0.96 | 0.96 | .053 |  |  | 112.433 | 43 | .000 | 0.91 | 0.91 | 0.066 |  |  | 148.47 | 43 | .000 | 0.86 | 0.85 | 0.082 |  |
| ∆ |  | 13.01 | 4 | .011 | -0.03 | -0.02 | .015 |  |  | 10.222 | 5 | .069 | -0.01 | 0.01 | -0.002 |  |  | 42.705 | 5 | .000 | -0.05 | -0.04 | 0.012 |  |

λ: factor loadings, τ = indicator intercepts, ε = indicator residual variances

Configural - Free λ; Weak (metric) - Equal λ; Strong (scalar) - Equal λ and τ; Strict - Equal λ, τ and ε

χ^2^: Chi-square test; df: degrees of freedom; p: p-value; CFI: Comparative Fit Index; TLI: Tucker Lewis Index; RMSEA: Root Mean Square Error of Approximation

∆: Difference between models
^a^ Readiness: 3 items, no residual covariances

^b^ Agency: 5 items, no residual covariances

^c^ Reflexivity: 5 items, no residual covariances estimated
