# Supplementary material for: The sensitivity of TANDEM – A new measure of trauma competence
Source: PLoS One. 2026 Jan 28;21(1):e0339858. doi: 10.1371/journal.pone.0339858 (PMC12851466; doi:10.1371/journal.pone.0339858)
Supplement: S2 Table — (DOCX) [file pone.0339858.s002.docx]

Supplementary Table 2: standardized factor loadings for
Readiness, Agency, and Reflexivity

| Indicators |  | Readiness | |  | Agency | |  | Reflexivity | |
| --- | --- | --- | --- | --- | --- | --- | --- | --- | --- |
|  |  | Pre | Post |  | Pre | Post |  | Pre | Post |
| 1 |  | .82 | .81 |  | .71 | .70 |  | .62 | .70 |
| 2 |  | .41 | .50 |  | .42 | .45 |  | .47 | .46 |
| 3 |  | .68 | .59 |  | .71 | .66 |  | .69 | .78 |
| 4 |  |  |  |  | .45 | .42 |  | .63 | .60 |
| 5 |  |  |  |  | .74 | .77 |  | .55 | .64 |
