# Supplementary material for: The sensitivity of TANDEM – A new measure of trauma competence
Source: PLoS One. 2026 Jan 28;21(1):e0339858. doi: 10.1371/journal.pone.0339858 (PMC12851466; doi:10.1371/journal.pone.0339858)
Supplement: S1 File — (PDF) [file pone.0339858.s003.pdf]

Codebook «Tandem sensitivity»

SkjemaID

		Value	Count	Percent
Standard Attributes	Position	1		
	Label	SkjemaID		
	Type	Numeric		
	Format	F8		
	Measurement	Nominal		
	Role	Input		
Valid Values	100027		98	26,5%
	100074		94	25,4%
	100105		81	21,9%
	100327		97	26,2%

PersonID

		Value
Standard Attributes	Position	2
	Label	PersonID
	Type	Numeric
	Format	F8
	Measurement	Nominal
	Role	Input

ARdy1

		Value	Count	Percent
Standard Attributes	Position	3		
	Label	A_Rdy1 = Jeg er positiv til å anvende en slik tilnærming i jobben min		
	Type	Numeric		
	Format	F8		
	Measurement	Ordinal		
	Role	Input		
Valid Values	1	Totally disagree	7	1,9%
	2	-	5	1,4%
	3	-	2	0,5%
	4	-	15	4,1%
	5	-	71	19,2%
	6	Totally agree	216	58,4%
Missing Values	-9999		54	14,6%

ARdy2

		Value	Count	Percent
Standard Attributes	Position	4		
	Label	A_Rdy2 = Det er en god tilnærming til dem med utfordrende atferd		
	Type	Numeric		

	Format	F8		
	Measurement	Ordinal		
	Role	Input		
Valid Values	1	Totally disagree	20	5,4%
	2	-	13	3,5%
	3	-	17	4,6%
	4	-	36	9,7%
	5	-	89	24,1%
	6	Totally agree	141	38,1%
Missing Values	-9999		54	14,6%

ARdy3

		Value	Count	Percent
Standard Attributes	Position	5		
	Label	A_Rdy3 = En slik tilnærming er nyttig for alle dem jeg møter i jobben		
	Type	Numeric		
	Format	F8		
	Measurement	Ordinal		
	Role	Input		
Valid Values	1	Totally disagree	3	0,8%
	2	-	11	3,0%
	3	-	27	7,3%
	4	-	46	12,4%
	5	-	87	23,5%
	6	Totally agree	142	38,4%
Missing Values	-9999		54	14,6%

AAgn1

		Value	Count	Percent
Standard Attributes	Position	6		
	Label	A_Agn1 = Jeg føler at jeg er i stand til å hjelpe dem		
	Type	Numeric		
	Format	F8		
	Measurement	Ordinal		
	Role	Input		
Valid Values	1	Rarely	2	0,5%
	2	-	17	4,6%
	3	-	69	18,6%
	4	-	124	33,5%
	5	-	73	19,7%
	6	Usually	30	8,1%
Missing Values	-9999		55	14,9%

AAgn2

		Value	Count	Percent
Standard Attributes	Position	7		
	Label	A_Agn2 = Jeg gruer meg for å jobbe med dem		
	Type	Numeric		
	Format	F8		

	Measurement	Ordinal		
	Role	Input		
Valid Values	1	Rarely	2	0,5%
	2	-	21	5,7%
	3	-	60	16,2%
	4	-	80	21,6%
	5	-	95	25,7%
	6	Usually	57	15,4%
Missing Values	-9999		55	14,9%

AAgn3

		Value	Count	Percent
Standard Attributes	Position	8		
	Label	A_Agn3 = Jeg opplever slikt arbeid som motiverende		
	Type	Numeric		
	Format	F8		
	Measurement	Ordinal		
	Role	Input		
Valid Values	1	Rarely	2	0,5%
	2	-	14	3,8%
	3	-	46	12,4%
	4	-	96	25,9%
	5	-	96	25,9%
	6	Usually	61	16,5%
Missing Values	-9999		55	14,9%

AAgn4

		Value	Count	Percent
Standard Attributes	Position	9		
	Label	A_Agn4 = Jeg mister troen på at det vil gå bra med dem		
	Type	Numeric		
	Format	F8		
	Measurement	Ordinal		
	Role	Input		
Valid Values	1	Rarely	0	0,0%
	2	-	19	5,1%
	3	-	52	14,1%
	4	-	97	26,2%
	5	-	105	28,4%
	6	Usually	42	11,4%
Missing Values	-9999		55	14,9%

AAgn5

		Value	Count	Percent
Standard Attributes	Position	10		
	Label	A_Agn5 = Jeg føler meg som en viktig hjelper for dem		
	Type	Numeric		
	Format	F8		
	Measurement	Ordinal		

	Role	Input		
Valid Values	1	Rarely	2	0,5%
	2	-	13	3,5%
	3	-	66	17,8%
	4	-	96	25,9%
	5	-	86	23,2%
	6	Usually	53	14,3%
Missing Values	-9999		54	14,6%

ARfx1

		Value	Count	Percent
Standard Attributes	Position	11		
	Label	A_Rfx1 = Hvordan stress i ditt eget liv påvirker deg i samspillet		
	Type	Numeric		
	Format	F8		
	Measurement	Ordinal		
	Role	Input		
Valid Values	1	To a small degree	8	2,2%
	2	-	23	6,2%
	3	-	29	7,8%
	4	-	87	23,5%
	5	-	88	23,8%
	6	To a great degree	79	21,4%
Missing Values	-9999		56	15,1%

ARfx2

		Value	Count	Percent
Standard Attributes	Position	12		
	Label	A_Rfx2 = Hvordan kolleger kan hjelpe deg å se din rolle i samspillet		
	Type	Numeric		
	Format	F8		
	Measurement	Ordinal		
	Role	Input		
Valid Values	1	To a small degree	6	1,6%
	2	-	17	4,6%
	3	-	33	8,9%
	4	-	65	17,6%
	5	-	119	32,2%
	6	To a great degree	73	19,7%
Missing Values	-9999		57	15,4%

ARfx3

		Value	Count	Percent
Standard Attributes	Position	13		
	Label	A_Rfx3 = Hvilke atferds- og følelsesuttrykk som trigger deg mest		

	Type	Numeric		
	Format	F8		
	Measurement	Ordinal		
	Role	Input		
Valid Values	1	To a small degree	4	1,1%
	2	-	16	4,3%
	3	-	36	9,7%
	4	-	90	24,3%
	5	-	106	28,6%
	6	To a great degree	59	15,9%
Missing Values	-9999		59	15,9%

ARfx4

		Value	Count	Percent
Standard Attributes	Position	14		
	Label	A_Rfx4 = Hvordan din egen oppvekst preger hva som trigger deg		
	Type	Numeric		
	Format	F8		
	Measurement	Ordinal		
	Role	Input		
Valid Values	1	To a small degree	20	5,4%
	2	-	34	9,2%
	3	-	44	11,9%
	4	-	80	21,6%
	5	-	83	22,4%
	6	To a great degree	53	14,3%
Missing Values	-9999		56	15,1%

ARfx5

		Value	Count	Percent
Standard Attributes	Position	15		
	Label	A_Rfx5 = Hva som kan oppleves truende ved din måte å være på		
	Type	Numeric		
	Format	F8		
	Measurement	Ordinal		
	Role	Input		
Valid Values	1	To a small degree	8	2,2%
	2	-	33	8,9%
	3	-	51	13,8%
	4	-	83	22,4%
	5	-	95	25,7%
	6	To a great degree	43	11,6%
Missing Values	-9999		57	15,4%

ARfx6

		Value	Count	Percent
Standard Attributes	Position	16		

	Label	A_Rfx6 = Hvordan du kan ivareta deg selv for å holde ut i arbeidet		
	Type	Numeric		
	Format	F8		
	Measurement	Ordinal		
	Role	Input		
Valid Values	1	To a small degree	7	1,9%
	2	-	39	10,5%
	3	-	67	18,1%
	4	-	79	21,4%
	5	-	68	18,4%
	6	To a great degree	54	14,6%
Missing Values	-9999		56	15,1%

ATkwl1

		Value	Count	Percent
Standard Attributes	Position	17		
	Label	A_Tkw1 = Hvordan tidlige samspillserfaringer påvirker barns utvikling		
	Type	Numeric		
	Format	F8		
	Measurement	Ordinal		
	Role	Input		
Valid Values	1	Not much	18	4,9%
	2	-	56	15,1%
	3	-	68	18,4%
	4	-	78	21,1%
	5	-	73	19,7%
	6	Very much	21	5,7%
Missing Values	-9999		56	15,1%

ATkw2

		Value	Count	Percent
Standard Attributes	Position	18		
	Label	A_Tkw2 = Hva som er sentrale utviklingsbehov i ulike utviklingsfaser		
	Type	Numeric		
	Format	F8		
	Measurement	Ordinal		
	Role	Input		
Valid Values	1	Not much	25	6,8%
	2	-	74	20,0%
	3	-	83	22,4%
	4	-	76	20,5%
	5	-	45	12,2%
	6	Very much	11	3,0%
Missing Values	-9999		56	15,1%

ATkw3

		Value	Count	Percent
Standard Attributes	Position	19		
	Label	A_Tkw3 = Hvordan traumer kan gi både for høyt og for lavt aktiveringsnivå		
	Type	Numeric		
	Format	F8		
	Measurement	Ordinal		
	Role	Input		
Valid Values	1	Not much	25	6,8%
	2	-	44	11,9%
	3	-	68	18,4%
	4	-	93	25,1%
	5	-	63	17,0%
	6	Very much	21	5,7%
Missing Values	-9999		56	15,1%

ATkw4

		Value	Count	Percent
Standard Attributes	Position	20		
	Label	A_Tkw4 = Hvordan traumer virker inn på hjernen og nervesystemet		
	Type	Numeric		
	Format	F8		
	Measurement	Ordinal		
	Role	Input		
Valid Values	1	Not much	38	10,3%
	2	-	60	16,2%
	3	-	71	19,2%
	4	-	82	22,2%
	5	-	44	11,9%
	6	Very much	19	5,1%
Missing Values	-9999		56	15,1%

ATkw5

		Value	Count	Percent
Standard Attributes	Position	21		
	Label	A_Tkw5 = Hvordan traumer påvirker kapasiteten til å lære og utforske		
	Type	Numeric		
	Format	F8		
	Measurement	Ordinal		
	Role	Input		
Valid Values	1	Not much	23	6,2%
	2	-	51	13,8%
	3	-	80	21,6%
	4	-	76	20,5%
	5	-	60	16,2%
	6	Very much	24	6,5%

Missing Values	-9999		56	15,1%

ATkw6

		Value	Count	Percent
Standard Attributes	Position	22		
	Label	A_Tkw6 = Hvilke konsekvenser traumer har for den fysiske helsen		
	Type	Numeric		
	Format	F8		
	Measurement	Ordinal		
	Role	Input		
Valid Values	1	Not much	20	5,4%
	2	-	49	13,2%
	3	-	72	19,5%
	4	-	83	22,4%
	5	-	63	17,0%
	6	Very much	27	7,3%
Missing Values	-9999		56	15,1%

ATkw7

		Value	Count	Percent
Standard Attributes	Position	23		
	Label	A_Tkw7 = Hva som skiller utviklingstraumer fra andre typer traumer		
	Type	Numeric		
	Format	F8		
	Measurement	Ordinal		
	Role	Input		
Valid Values	1	Not much	44	11,9%
	2	-	97	26,2%
	3	-	68	18,4%
	4	-	64	17,3%
	5	-	32	8,6%
	6	Very much	8	2,2%
Missing Values	-9999		57	15,4%

ATkw8

		Value	Count	Percent
Standard Attributes	Position	24		
	Label	A_Tkw8 = Hvordan traumereaksjoner påvirkes av kulturell kontekst		
	Type	Numeric		
	Format	F8		
	Measurement	Ordinal		
	Role	Input		
Valid Values	1	Not much	50	13,5%
	2	-	92	24,9%
	3	-	89	24,1%
	4	-	49	13,2%

	5	-	25	6,8%
	6	Very much	9	2,4%
Missing Values	-9999		56	15,1%

ACkw1

		Value	Count	Percent
Standard Attributes	Position	25		
	Label	A_Ckw1 = Reguleringsstøtte		
	Type	Numeric		
	Format	F8		
	Measurement	Ordinal		
	Role	Input		
Valid Values	1	Not much	48	13,0%
	2	-	73	19,7%
	3	-	59	15,9%
	4	-	80	21,6%
	5	-	39	10,5%
	6	Very much	14	3,8%
Missing Values	-9999		57	15,4%

ACkw2

		Value	Count	Percent
Standard Attributes	Position	26		
	Label	A_Ckw2 = Reguleringsvansker		
	Type	Numeric		
	Format	F8		
	Measurement	Ordinal		
	Role	Input		
Valid Values	1	Not much	28	7,6%
	2	-	55	14,9%
	3	-	70	18,9%
	4	-	86	23,2%
	5	-	56	15,1%
	6	Very much	16	4,3%
Missing Values	-9999		59	15,9%

ACkw3

		Value	Count	Percent
Standard Attributes	Position	27		
	Label	A_Ckw3 = Toleransevinduet		
	Type	Numeric		
	Format	F8		
	Measurement	Ordinal		
	Role	Input		
Valid Values	1	Not much	21	5,7%
	2	-	28	7,6%
	3	-	55	14,9%
	4	-	77	20,8%
	5	-	82	22,2%
	6	Very much	50	13,5%
Missing Values	-9999		57	15,4%

ACkw4

		Value	Count	Percent

Standard Attributes	Position	28		
	Label	A_Ckw4 = Traumetriggere		
	Type	Numeric		
	Format	F8		
	Measurement	Ordinal		
	Role	Input		
Valid Values	1	Not much	23	6,2%
	2	-	51	13,8%
	3	-	69	18,6%
	4	-	84	22,7%
	5	-	65	17,6%
	6	Very much	21	5,7%
Missing Values	-9999		57	15,4%

ACkw5

		Value	Count	Percent
Standard Attributes	Position	29		
	Label	A_Ckw5 = Dissosiasjon		
	Type	Numeric		
	Format	F8		
	Measurement	Ordinal		
	Role	Input		
Valid Values	1	Not much	30	8,1%
	2	-	39	10,5%
	3	-	81	21,9%
	4	-	89	24,1%
	5	-	53	14,3%
	6	Very much	22	5,9%
Missing Values	-9999		56	15,1%

ACkw6

		Value	Count	Percent
Standard Attributes	Position	30		
	Label	A_Ckw6 = Posttraumatisk stresslidelse		
	Type	Numeric		
	Format	F8		
	Measurement	Ordinal		
	Role	Input		
Valid Values	1	Not much	12	3,2%
	2	-	35	9,5%
	3	-	69	18,6%
	4	-	104	28,1%
	5	-	68	18,4%
	6	Very much	26	7,0%
Missing Values	-9999		56	15,1%

ASus1

		Value	Count	Percent
Standard Attributes	Position	31		
	Label	A_Sus1 = Finne aktiviteter som er passe utfordrende for dem?		

	Type	Numeric		
	Format	F8		
	Measurement	Ordinal		
	Role	Input		
Valid Values	1	To a small degree	27	7,3%
	2	-	65	17,6%
	3	-	89	24,1%
	4	-	93	25,1%
	5	-	37	10,0%
	6	To a great degree	3	0,8%
Missing Values	-9999		56	15,1%

ASus2

		Value	Count	Percent
Standard Attributes	Position	32		
	Label	A_Sus2 = Mngment5_Plan Regulere deres stressnivå med ulike typer sansestimulering ?		
	Type	Numeric		
	Format	F8		
	Measurement	Ordinal		
	Role	Input		
Valid Values	1	To a small degree	40	10,8%
	2	-	86	23,2%
	3	-	88	23,8%
	4	-	66	17,8%
	5	-	30	8,1%
	6	To a great degree	3	0,8%
Missing Values	-9999		57	15,4%

ASus3

		Value	Count	Percent
Standard Attributes	Position	33		
	Label	A_Sus3 = Snakke med dem for å avdekke traumeerfaringer ?		
	Type	Numeric		
	Format	F8		
	Measurement	Ordinal		
	Role	Input		
Valid Values	1	To a small degree	35	9,5%
	2	-	86	23,2%
	3	-	84	22,7%
	4	-	65	17,6%
	5	-	38	10,3%
	6	To a great degree	6	1,6%
Missing Values	-9999		56	15,1%

ASus4

		Value	Count	Percent
Standard Attributes	Position	34		
	Label	A_Sus4 = Snakke med dem om traumeerfaringer du kjenner til at de har?		
	Type	Numeric		
	Format	F8		
	Measurement	Ordinal		
	Role	Input		
Valid Values	1	To a small degree	25	6,8%
	2	-	78	21,1%
	3	-	78	21,1%
	4	-	82	22,2%
	5	-	42	11,4%
	6	To a great degree	8	2,2%
Missing Values	-9999		57	15,4%

ASus5

		Value	Count	Percent
Standard Attributes	Position	35		
	Label	A_Sus5 = Hjelpe dem å forstå hvorfor de reagerer som de gjør?		
	Type	Numeric		
	Format	F8		
	Measurement	Ordinal		
	Role	Input		
Valid Values	1	To a small degree	24	6,5%
	2	-	73	19,7%
	3	-	86	23,2%
	4	-	90	24,3%
	5	-	37	10,0%
	6	To a great degree	4	1,1%
Missing Values	-9999		56	15,1%

ASus6

		Value	Count	Percent
Standard Attributes	Position	36		
	Label	A_Sus6 = Snakke med dem på en måte som gir fremtidshåp?		
	Type	Numeric		
	Format	F8		
	Measurement	Ordinal		
	Role	Input		
Valid Values	1	To a small degree	10	2,7%
	2	-	31	8,4%
	3	-	79	21,4%
	4	-	109	29,5%

	5	-	74	20,0%
	6	To a great degree	11	3,0%
Missing Values	-9999		56	15,1%

ASus7

		Value	Count	Percent
Standard Attributes	Position	37		
	Label	A_Sus7 = Involvere familie og andre i et støttende nettverk rundt dem?		
	Type	Numeric		
	Format	F8		
	Measurement	Ordinal		
	Role	Input		
Valid Values	1	To a small degree	31	8,4%
	2	-	65	17,6%
	3	-	78	21,1%
	4	-	79	21,4%
	5	-	46	12,4%
	6	To a great degree	15	4,1%
Missing Values	-9999		56	15,1%

ASus8

		Value	Count	Percent
Standard Attributes	Position	38		
	Label	A_Sus8 = Tilpasse hjelpen til deres egen kulturelle forståelse?		
	Type	Numeric		
	Format	F8		
	Measurement	Ordinal		
	Role	Input		
Valid Values	1	To a small degree	56	15,1%
	2	-	86	23,2%
	3	-	89	24,1%
	4	-	55	14,9%
	5	-	24	6,5%
	6	To a great degree	4	1,1%
Missing Values	-9999		56	15,1%

ASis1

		Value	Count	Percent
Standard Attributes	Position	39		
	Label	A_Sis1 = Møte dem hvis de lukker seg inn i seg selv?		
	Type	Numeric		
	Format	F8		
	Measurement	Ordinal		
	Role	Input		

Valid Values	1	To a small degree	35	9,5%
	2	-	68	18,4%
	3	-	91	24,6%
	4	-	78	21,1%
	5	-	35	9,5%
	6	To a great degree	7	1,9%
Missing Values	-9999		56	15,1%

ASis2

		Value	Count	Percent
Standard Attributes	Position	40		
	Label	A_Sis2 = Avveie når du bør være nær, og når du bør gi rom?		
	Type	Numeric		
	Format	F8		
	Measurement	Ordinal		
	Role	Input		
Valid Values	1	To a small degree	11	3,0%
	2	-	45	12,2%
	3	-	86	23,2%
	4	-	102	27,6%
	5	-	50	13,5%
	6	To a great degree	20	5,4%
Missing Values	-9999		56	15,1%

ASis3

		Value	Count	Percent
Standard Attributes	Position	41		
	Label	A_Sis3 = Avveie når de bør stoppes fra noe de forsøker å gjøre?		
	Type	Numeric		
	Format	F8		
	Measurement	Ordinal		
	Role	Input		
Valid Values	1	To a small degree	14	3,8%
	2	-	52	14,1%
	3	-	82	22,2%
	4	-	103	27,8%
	5	-	50	13,5%
	6	To a great degree	13	3,5%
Missing Values	-9999		56	15,1%

ASis4

		Value	Count	Percent
Standard Attributes	Position	42		
	Label	A_Sis4 = Avveie når de bør utfordres til å gjøre noe de motsetter seg?		
	Type	Numeric		

	Format	F8		
	Measurement	Ordinal		
	Role	Input		
Valid Values	1	To a small degree	18	4,9%
	2	-	46	12,4%
	3	-	99	26,8%
	4	-	99	26,8%
	5	-	45	12,2%
	6	To a great degree	6	1,6%
Missing Values	-9999		57	15,4%

ASis5

		Value	Count	Percent
Standard Attributes	Position	43		
	Label	A_Sis5 = Møte dem hvis de blir verbalt utagerende?		
	Type	Numeric		
	Format	F8		
	Measurement	Ordinal		
	Role	Input		
Valid Values	1	To a small degree	18	4,9%
	2	-	36	9,7%
	3	-	59	15,9%
	4	-	104	28,1%
	5	-	65	17,6%
	6	To a great degree	32	8,6%
Missing Values	-9999		56	15,1%

ASis6

		Value	Count	Percent
Standard Attributes	Position	44		
	Label	A_Sis6 = Møte dem hvis de blir fysisk utagerende?		
	Type	Numeric		
	Format	F8		
	Measurement	Ordinal		
	Role	Input		
Valid Values	1	To a small degree	33	8,9%
	2	-	59	15,9%
	3	-	65	17,6%
	4	-	74	20,0%
	5	-	54	14,6%
	6	To a great degree	29	7,8%
Missing Values	-9999		56	15,1%

ASis7

		Value	Count	Percent
Standard Attributes	Position	45		
	Label	A_Sis7 = Regulere ditt eget stressnivå		

		når du føler deg utfordret?		
	Type	Numeric		
	Format	F8		
	Measurement	Ordinal		
	Role	Input		
Valid Values	1	To a small degree	12	3,2%
	2	-	38	10,3%
	3	-	67	18,1%
	4	-	106	28,6%
	5	-	69	18,6%
	6	To a great degree	22	5,9%
Missing Values	-9999		56	15,1%

AUsc1

		Value	Count	Percent
Standard Attributes	Position	46		
	Label	A_Usc1 = Vi har en felles forståelse for hvordan vi bør møte dem		
	Type	Numeric		
	Format	F8		
	Measurement	Ordinal		
	Role	Input		
Valid Values	1	To a small degree	26	7,0%
	2	-	42	11,4%
	3	-	68	18,4%
	4	-	102	27,6%
	5	-	63	17,0%
	6	To a great degree	12	3,2%
Missing Values	-9999		57	15,4%

AUsc2

		Value	Count	Percent
Standard Attributes	Position	47		
	Label	A_Usc2 = Vi er bevisst på hvordan vi omtaler dem oss imellom		
	Type	Numeric		
	Format	F8		
	Measurement	Ordinal		
	Role	Input		
Valid Values	1	To a small degree	19	5,1%
	2	-	43	11,6%
	3	-	81	21,9%
	4	-	89	24,1%
	5	-	63	17,0%
	6	To a great degree	18	4,9%
Missing Values	-9999		57	15,4%

AUsc3

		Value	Count	Percent
Standard Attributes	Position	48		
	Label	A_Usc3 = Vi undersøker om de lever med pågående belastninger		
	Type	Numeric		
	Format	F8		
	Measurement	Ordinal		
	Role	Input		
Valid Values	1	To a small degree	16	4,3%
	2	-	39	10,5%
	3	-	66	17,8%
	4	-	80	21,6%
	5	-	80	21,6%
	6	To a great degree	31	8,4%
Missing Values	-9999		58	15,7%

AUsc4

		Value	Count	Percent
Standard Attributes	Position	49		
	Label	A_Usc4 = Vi hjelper hverandre til å se deres fremtidsmulighet er		
	Type	Numeric		
	Format	F8		
	Measurement	Ordinal		
	Role	Input		
Valid Values	1	To a small degree	6	1,6%
	2	-	29	7,8%
	3	-	64	17,3%
	4	-	109	29,5%
	5	-	80	21,6%
	6	To a great degree	25	6,8%
Missing Values	-9999		57	15,4%

AUsc5

		Value	Count	Percent
Standard Attributes	Position	50		
	Label	A_Usc5 = Vi jobber for å forebygge at de utsetter seg for risiko		
	Type	Numeric		
	Format	F8		
	Measurement	Ordinal		
	Role	Input		
Valid Values	1	To a small degree	9	2,4%
	2	-	19	5,1%
	3	-	49	13,2%
	4	-	90	24,3%

	5	-	99	26,8%
	6	To a great degree	48	13,0%
Missing Values	-9999		56	15,1%

AUsc6

		Value	Count	Percent
Standard Attributes	Position	51		
	Label	A_Usc6 = Vi har rutiner som gir dem trygghet og forutsigbarhet		
	Type	Numeric		
	Format	F8		
	Measurement	Ordinal		
	Role	Input		
Valid Values	1	To a small degree	12	3,2%
	2	-	24	6,5%
	3	-	48	13,0%
	4	-	90	24,3%
	5	-	88	23,8%
	6	To a great degree	52	14,1%
Missing Values	-9999		56	15,1%

ACsc1

		Value	Count	Percent
Standard Attributes	Position	52		
	Label	A_Csc1 = Vi samarbeider med andre tjenester om tiltakene		
	Type	Numeric		
	Format	F8		
	Measurement	Ordinal		
	Role	Input		
Valid Values	1	To a small degree	6	1,6%
	2	-	26	7,0%
	3	-	60	16,2%
	4	-	84	22,7%
	5	-	86	23,2%
	6	To a great degree	50	13,5%
Missing Values	-9999		58	15,7%

ACsc2

		Value	Count	Percent
Standard Attributes	Position	53		
	Label	A_Csc2 = Vi snakker om hvordan vi selv påvirker samspillet		
	Type	Numeric		
	Format	F8		
	Measurement	Ordinal		
	Role	Input		
Valid Values	1	To a small degree	17	4,6%

	2	-	49	13,2%
	3	-	78	21,1%
	4	-	81	21,9%
	5	-	64	17,3%
	6	To a great degree	22	5,9%
Missing Values	-9999		59	15,9%

ACsc3

		Value	Count	Percent
Standard Attributes	Position	54		
	Label	A_Csc3 = Vi har åpenhet om situasjoner vi syns er vanskelige		
	Type	Numeric		
	Format	F8		
	Measurement	Ordinal		
	Role	Input		
Valid Values	1	To a small degree	5	1,4%
	2	-	9	2,4%
	3	-	49	13,2%
	4	-	51	13,8%
	5	-	109	29,5%
	6	To a great degree	89	24,1%
Missing Values	-9999		58	15,7%

ACsc4

		Value	Count	Percent
Standard Attributes	Position	55		
	Label	A_Csc4 = Vi fremhever viktigheten av å ha traumekunnskap		
	Type	Numeric		
	Format	F8		
	Measurement	Ordinal		
	Role	Input		
Valid Values	1	To a small degree	35	9,5%
	2	-	67	18,1%
	3	-	76	20,5%
	4	-	61	16,5%
	5	-	48	13,0%
	6	To a great degree	23	6,2%
Missing Values	-9999		60	16,2%

ACsc5

		Value	Count	Percent
Standard Attributes	Position	56		
	Label	A_Csc5 = Vi er opptatt av å jobbe kultursensitivt		
	Type	Numeric		
	Format	F8		

	Measurement	Ordinal		
	Role	Input		
Valid Values	1	To a small degree	41	11,1%
	2	-	61	16,5%
	3	-	71	19,2%
	4	-	71	19,2%
	5	-	48	13,0%
	6	To a great degree	19	5,1%
Missing Values	-9999		59	15,9%

ACsc6

		Value	Count	Percent
Standard Attributes	Position	57		
	Label	A_Csc6 = Vi får veiledning på arbeid med utfordrende saker		
	Type	Numeric		
	Format	F8		
	Measurement	Ordinal		
	Role	Input		
Valid Values	1	To a small degree	43	11,6%
	2	-	59	15,9%
	3	-	45	12,2%
	4	-	69	18,6%
	5	-	62	16,8%
	6	To a great degree	34	9,2%
Missing Values	-9999		58	15,7%

BRdy1

		Value	Count	Percent
Standard Attributes	Position	58		
	Label	B_Rdy1 = Jeg er positiv til å anvende en slik tilnærming i jobben min		
	Type	Numeric		
	Format	F8		
	Measurement	Ordinal		
	Role	Input		
Valid Values	1	Totally disagree	3	0,8%
	2	-	0	0,0%
	3	-	1	0,3%
	4	-	5	1,4%
	5	-	48	13,0%
	6	Totally agree	184	49,7%
Missing Values	-9999		129	34,9%

BRdy2

		Value	Count	Percent
Standard Attributes	Position	59		
	Label	B_Rdy2 = Det er en god tilnærming til dem med		

		utfordrende atferd		
	Type	Numeric		
	Format	F8		
	Measurement	Ordinal		
	Role	Input		
Valid Values	1	Totally disagree	12	3,2%
	2	-	8	2,2%
	3	-	10	2,7%
	4	-	17	4,6%
	5	-	65	17,6%
	6	Totally agree	127	34,3%
Missing Values	-9999		131	35,4%

BRdy3

		Value	Count	Percent
Standard Attributes	Position	60		
	Label	B_Rdy3 = En slik tilnærming er nyttig for alle dem jeg møter i jobben		
	Type	Numeric		
	Format	F8		
	Measurement	Ordinal		
	Role	Input		
Valid Values	1	Totally disagree	7	1,9%
	2	-	2	0,5%
	3	-	11	3,0%
	4	-	33	8,9%
	5	-	71	19,2%
	6	Totally agree	116	31,4%
Missing Values	-9999		130	35,1%

BAgn1

		Value	Count	Percent
Standard Attributes	Position	61		
	Label	B_Agn1 = Jeg føler at jeg er i stand til å hjelpe dem		
	Type	Numeric		
	Format	F8		
	Measurement	Ordinal		
	Role	Input		
Valid Values	1	Rarely	1	0,3%
	2	-	4	1,1%
	3	-	24	6,5%
	4	-	110	29,7%
	5	-	74	20,0%
	6	Usually	26	7,0%
Missing Values	-9999		131	35,4%

BAgn2

		Value	Count	Percent
Standard Attributes	Position	62		
	Label	B_Agn2 = Jeg gruer meg for å		

		jobbe med dem		
	Type	Numeric		
	Format	F8		
	Measurement	Ordinal		
	Role	Input		
Valid Values	1	Rarely	2	0,5%
	2	-	14	3,8%
	3	-	50	13,5%
	4	-	44	11,9%
	5	-	88	23,8%
	6	Usually	40	10,8%
Missing Values	-9999		132	35,7%

BAgn3

		Value	Count	Percent
Standard Attributes	Position	63		
	Label	B_Agn3 = Jeg opplever slikt arbeid som motiverende		
	Type	Numeric		
	Format	F8		
	Measurement	Ordinal		
	Role	Input		
Valid Values	1	Rarely	0	0,0%
	2	-	2	0,5%
	3	-	27	7,3%
	4	-	65	17,6%
	5	-	92	24,9%
	6	Usually	53	14,3%
Missing Values	-9999		131	35,4%

BAgn4

		Value	Count	Percent
Standard Attributes	Position	64		
	Label	B_Agn4 = Jeg mister troen på at det vil gå bra med dem		
	Type	Numeric		
	Format	F8		
	Measurement	Ordinal		
	Role	Input		
Valid Values	1	Rarely	1	0,3%
	2	-	12	3,2%
	3	-	30	8,1%
	4	-	62	16,8%
	5	-	97	26,2%
	6	Usually	36	9,7%
Missing Values	-9999		132	35,7%

BAgn5

		Value	Count	Percent
Standard Attributes	Position	65		
	Label	B_Agn5 = Jeg føler meg som en viktig hjelper for dem		

	Type	Numeric		
	Format	F8		
	Measurement	Ordinal		
	Role	Input		
Valid Values	1	Rarely	2	0,5%
	2	-	5	1,4%
	3	-	30	8,1%
	4	-	73	19,7%
	5	-	82	22,2%
	6	Usually	48	13,0%
Missing Values	-9999		130	35,1%

BRfx1

		Value	Count	Percent
Standard Attributes	Position	66		
	Label	B_Rfx1 = Hvordan stress i ditt eget liv påvirker deg i samspillet		
	Type	Numeric		
	Format	F8		
	Measurement	Ordinal		
	Role	Input		
Valid Values	1	To a small degree	3	0,8%
	2	-	12	3,2%
	3	-	19	5,1%
	4	-	42	11,4%
	5	-	84	22,7%
	6	To a great degree	79	21,4%
Missing Values	-9999		131	35,4%

BRfx2

		Value	Count	Percent
Standard Attributes	Position	67		
	Label	B_Rfx2 = Hvordan kolleger kan hjelpe deg å se din rolle i samspillet		
	Type	Numeric		
	Format	F8		
	Measurement	Ordinal		
	Role	Input		
Valid Values	1	To a small degree	1	0,3%
	2	-	3	0,8%
	3	-	10	2,7%
	4	-	57	15,4%
	5	-	101	27,3%
	6	To a great degree	65	17,6%
Missing Values	-9999		133	35,9%

BRfx3

		Value	Count	Percent
Standard Attributes	Position	68		
	Label	B_Rfx3 = Hvilke		

		atferds- og følelsesuttrykk som trigger deg mest		
	Type	Numeric		
	Format	F8		
	Measurement	Ordinal		
	Role	Input		
Valid Values	1	To a small degree	0	0,0%
	2	-	8	2,2%
	3	-	19	5,1%
	4	-	62	16,8%
	5	-	87	23,5%
	6	To a great degree	62	16,8%
Missing Values	-9999		132	35,7%

BRfx4

		Value	Count	Percent
Standard Attributes	Position	69		
	Label	B_Rfx4_Hvordan din egen oppvekst preger hva som trigger deg		
	Type	Numeric		
	Format	F8		
	Measurement	Ordinal		
	Role	Input		
Valid Values	1	To a small degree	11	3,0%
	2	-	17	4,6%
	3	-	33	8,9%
	4	-	45	12,2%
	5	-	74	20,0%
	6	To a great degree	59	15,9%
Missing Values	-9999		131	35,4%

BRfx5

		Value	Count	Percent
Standard Attributes	Position	70		
	Label	B_Rfx5 = Hva som kan oppleves truende ved din måte å være på		
	Type	Numeric		
	Format	F8		
	Measurement	Ordinal		
	Role	Input		
Valid Values	1	To a small degree	1	0,3%
	2	-	14	3,8%
	3	-	26	7,0%
	4	-	62	16,8%
	5	-	90	24,3%
	6	To a great degree	45	12,2%
Missing Values	-9999		132	35,7%

BRfx6

		Value	Count	Percent
Standard Attributes	Position	71		
	Label	B_Rfx6 = Hvordan du kan ivareta deg selv for å holde ut i arbeidet		
	Type	Numeric		
	Format	F8		
	Measurement	Ordinal		
	Role	Input		
Valid Values	1	To a small degree	2	0,5%
	2	-	16	4,3%
	3	-	35	9,5%
	4	-	65	17,6%
	5	-	76	20,5%
	6	To a great degree	45	12,2%
Missing Values	-9999		131	35,4%

BTkw1

		Value	Count	Percent
Standard Attributes	Position	72		
	Label	B_Tkw1 = Hvordan tidlige samspillserfaringer påvirker barns utvikling		
	Type	Numeric		
	Format	F8		
	Measurement	Ordinal		
	Role	Input		
Valid Values	1	Not much	1	0,3%
	2	-	3	0,8%
	3	-	14	3,8%
	4	-	60	16,2%
	5	-	123	33,2%
	6	Very much	38	10,3%
Missing Values	-9999		131	35,4%

BTkw2

		Value	Count	Percent
Standard Attributes	Position	73		
	Label	B_Tkw2 = Hva som er sentrale utviklingsbehov i ulike utviklingsfaser		
	Type	Numeric		
	Format	F8		
	Measurement	Ordinal		
	Role	Input		
Valid Values	1	Not much	3	0,8%
	2	-	17	4,6%
	3	-	37	10,0%
	4	-	83	22,4%

	5	-	83	22,4%
	6	Very much	16	4,3%
Missing Values	-9999		131	35,4%

BTkw3

		Value	Count	Percent
Standard Attributes	Position	74		
	Label	B_Tkw3 = Hvordan traumer kan gi både for høyt og for lavt aktiveringsnivå		
	Type	Numeric		
	Format	F8		
	Measurement	Ordinal		
	Role	Input		
Valid Values	1	Not much	1	0,3%
	2	-	0	0,0%
	3	-	8	2,2%
	4	-	42	11,4%
	5	-	127	34,3%
	6	Very much	60	16,2%
Missing Values	-9999		132	35,7%

BTkw4

		Value	Count	Percent
Standard Attributes	Position	75		
	Label	B_Tkw4 = Hvordan traumer virker inn på hjernen og nervesystemet		
	Type	Numeric		
	Format	F8		
	Measurement	Ordinal		
	Role	Input		
Valid Values	1	Not much	0	0,0%
	2	-	0	0,0%
	3	-	18	4,9%
	4	-	75	20,3%
	5	-	106	28,6%
	6	Very much	40	10,8%
Missing Values	-9999		131	35,4%

BTkw5

		Value	Count	Percent
Standard Attributes	Position	76		
	Label	B_Tkw5 = Hvordan traumer påvirker kapasiteten til å lære og utforske		
	Type	Numeric		
	Format	F8		
	Measurement	Ordinal		
	Role	Input		
Valid Values	1	Not much	0	0,0%
	2	-	2	0,5%

	3	-	13	3,5%
	4	-	58	15,7%
	5	-	119	32,2%
	6	Very much	47	12,7%
Missing Values	-9999		131	35,4%

BTkw6

		Value	Count	Percent
Standard Attributes	Position	77		
	Label	B_Tkw6 = Hvilke konsekvenser traumer har for den fysiske helsen		
	Type	Numeric		
	Format	F8		
	Measurement	Ordinal		
	Role	Input		
Valid Values	1	Not much	0	0,0%
	2	-	1	0,3%
	3	-	21	5,7%
	4	-	66	17,8%
	5	-	119	32,2%
	6	Very much	32	8,6%
Missing Values	-9999		131	35,4%

BTkw7

		Value	Count	Percent
Standard Attributes	Position	78		
	Label	B_Tkw7 = Hva som skiller utviklingstraumer fra andre typer traumer		
	Type	Numeric		
	Format	F8		
	Measurement	Ordinal		
	Role	Input		
Valid Values	1	Not much	1	0,3%
	2	-	6	1,6%
	3	-	16	4,3%
	4	-	69	18,6%
	5	-	112	30,3%
	6	Very much	34	9,2%
Missing Values	-9999		132	35,7%

BTkw8

		Value	Count	Percent
Standard Attributes	Position	79		
	Label	B_Tkw8 = Hvordan traumereaksjoner påvirkes av kulturell kontekst		
	Type	Numeric		
	Format	F8		
	Measurement	Ordinal		
	Role	Input		

Valid Values	1	Not much	8	2,2%
	2	-	12	3,2%
	3	-	36	9,7%
	4	-	101	27,3%
	5	-	70	18,9%
	6	Very much	9	2,4%
Missing Values	-9999		134	36,2%

BCkw1

		Value	Count	Percent
Standard Attributes	Position	80		
	Label	B_Ckw1 = Reguleringsstøtte		
	Type	Numeric		
	Format	F8		
	Measurement	Ordinal		
	Role	Input		
Valid Values	1	Not much	3	0,8%
	2	-	1	0,3%
	3	-	20	5,4%
	4	-	71	19,2%
	5	-	114	30,8%
	6	Very much	30	8,1%
Missing Values	-9999		131	35,4%

BCkw2

		Value	Count	Percent
Standard Attributes	Position	81		
	Label	B_Ckw2 = Reguleringsvans ker		
	Type	Numeric		
	Format	F8		
	Measurement	Ordinal		
	Role	Input		
Valid Values	1	Not much	0	0,0%
	2	-	1	0,3%
	3	-	17	4,6%
	4	-	69	18,6%
	5	-	117	31,6%
	6	Very much	34	9,2%
Missing Values	-9999		132	35,7%

BCkw3

		Value	Count	Percent
Standard Attributes	Position	82		
	Label	B_Ckw3 = Toleransevinduet		
	Type	Numeric		
	Format	F8		
	Measurement	Ordinal		
	Role	Input		
Valid Values	1	Not much	0	0,0%
	2	-	0	0,0%
	3	-	6	1,6%
	4	-	34	9,2%
	5	-	118	31,9%
	6	Very much	81	21,9%

Missing Values	-9999		131	35,4%

BCkw4

		Value	Count	Percent
Standard Attributes	Position	83		
	Label	B_Ckw4 = Traumetriggere		
	Type	Numeric		
	Format	F8		
	Measurement	Ordinal		
	Role	Input		
Valid Values	1	Not much	1	0,3%
	2	-	0	0,0%
	3	-	11	3,0%
	4	-	67	18,1%
	5	-	117	31,6%
	6	Very much	43	11,6%
Missing Values	-9999		131	35,4%

BCkw5

		Value	Count	Percent
Standard Attributes	Position	84		
	Label	B_Ckw5 = Dissosiasjon		
	Type	Numeric		
	Format	F8		
	Measurement	Ordinal		
	Role	Input		
Valid Values	1	Not much	4	1,1%
	2	-	11	3,0%
	3	-	46	12,4%
	4	-	72	19,5%
	5	-	82	22,2%
	6	Very much	24	6,5%
Missing Values	-9999		131	35,4%

BCkw6

		Value	Count	Percent
Standard Attributes	Position	85		
	Label	B_Ckw6 = Posttraumatisk stresslidelse		
	Type	Numeric		
	Format	F8		
	Measurement	Ordinal		
	Role	Input		
Valid Values	1	Not much	1	0,3%
	2	-	3	0,8%
	3	-	24	6,5%
	4	-	76	20,5%
	5	-	100	27,0%
	6	Very much	34	9,2%
Missing Values	-9999		132	35,7%

BSus1

		Value	Count	Percent
Standard Attributes	Position	86		
	Label	B_Sus1 = Finne		

		aktiviteter som er passe utfordrende for dem?		
	Type	Numeric		
	Format	F8		
	Measurement	Ordinal		
	Role	Input		
Valid Values	1	To a small degree	3	0,8%
	2	-	13	3,5%
	3	-	48	13,0%
	4	-	108	29,2%
	5	-	56	15,1%
	6	To a great degree	8	2,2%
Missing Values	-9999		134	36,2%

BSus2

		Value	Count	Percent
Standard Attributes	Position	87		
	Label	B_Sus2 = Regulere deres stressnivå med ulike typer sansestimulering ?		
	Type	Numeric		
	Format	F8		
	Measurement	Ordinal		
	Role	Input		
Valid Values	1	To a small degree	2	0,5%
	2	-	11	3,0%
	3	-	41	11,1%
	4	-	102	27,6%
	5	-	69	18,6%
	6	To a great degree	12	3,2%
Missing Values	-9999		133	35,9%

BSus3

		Value	Count	Percent
Standard Attributes	Position	88		
	Label	B_Sus3 = Snakke med dem for å avdekke traumeerfaringer ?		
	Type	Numeric		
	Format	F8		
	Measurement	Ordinal		
	Role	Input		
Valid Values	1	To a small degree	5	1,4%
	2	-	14	3,8%
	3	-	45	12,2%
	4	-	101	27,3%
	5	-	60	16,2%
	6	To a great degree	12	3,2%
Missing Values	-9999		133	35,9%

BSus4

		Value	Count	Percent
Standard Attributes	Position	89		
	Label	B_Sus4 = Snakke med dem om traumeerfaringer du kjenner til at de har?		
	Type	Numeric		
	Format	F8		
	Measurement	Ordinal		
	Role	Input		
Valid Values	1	To a small degree	3	0,8%
	2	-	10	2,7%
	3	-	30	8,1%
	4	-	108	29,2%
	5	-	70	18,9%
	6	To a great degree	16	4,3%
Missing Values	-9999		133	35,9%

BSus5

		Value	Count	Percent
Standard Attributes	Position	90		
	Label	B_Sus5 = Hjelpe dem å forstå hvorfor de reagerer som de gjør?		
	Type	Numeric		
	Format	F8		
	Measurement	Ordinal		
	Role	Input		
Valid Values	1	To a small degree	1	0,3%
	2	-	8	2,2%
	3	-	23	6,2%
	4	-	97	26,2%
	5	-	90	24,3%
	6	To a great degree	17	4,6%
Missing Values	-9999		134	36,2%

BSus6

		Value	Count	Percent
Standard Attributes	Position	91		
	Label	B_Sus6 = Snakke med dem på en måte som gir fremtidshåp?		
	Type	Numeric		
	Format	F8		
	Measurement	Ordinal		
	Role	Input		
Valid Values	1	To a small degree	1	0,3%
	2	-	3	0,8%

	3	-	27	7,3%
	4	-	82	22,2%
	5	-	102	27,6%
	6	To a great degree	22	5,9%
Missing Values	-9999		133	35,9%

BSus7

		Value	Count	Percent
Standard Attributes	Position	92		
	Label	B_Sus7 = Involvere familie og andre i et støttende nettverk rundt dem?		
	Type	Numeric		
	Format	F8		
	Measurement	Ordinal		
	Role	Input		
Valid Values	1	To a small degree	3	0,8%
	2	-	9	2,4%
	3	-	44	11,9%
	4	-	89	24,1%
	5	-	67	18,1%
	6	To a great degree	24	6,5%
Missing Values	-9999		134	36,2%

BSus8

		Value	Count	Percent
Standard Attributes	Position	93		
	Label	B_Sus8 = Tilpasse hjelpen til deres egen kulturelle forståelse?		
	Type	Numeric		
	Format	F8		
	Measurement	Ordinal		
	Role	Input		
Valid Values	1	To a small degree	8	2,2%
	2	-	15	4,1%
	3	-	67	18,1%
	4	-	99	26,8%
	5	-	43	11,6%
	6	To a great degree	5	1,4%
Missing Values	-9999		133	35,9%

BSis1

		Value	Count	Percent
Standard Attributes	Position	94		
	Label	B_Sis1 = Møte dem hvis de lukker seg inn i seg selv?		
	Type	Numeric		
	Format	F8		

	Measurement	Ordinal		
	Role	Input		
Valid Values	1	To a small degree	4	1,1%
	2	-	19	5,1%
	3	-	55	14,9%
	4	-	100	27,0%
	5	-	50	13,5%
	6	To a great degree	8	2,2%
Missing Values	-9999		134	36,2%

BSis2

		Value	Count	Percent
Standard Attributes	Position	95		
	Label	B_Sis2 = Avveie når du bør være nær, og når du bør gi rom?		
	Type	Numeric		
	Format	F8		
	Measurement	Ordinal		
	Role	Input		
Valid Values	1	To a small degree	3	0,8%
	2	-	9	2,4%
	3	-	29	7,8%
	4	-	100	27,0%
	5	-	76	20,5%
	6	To a great degree	20	5,4%
Missing Values	-9999		133	35,9%

BSis3

		Value	Count	Percent
Standard Attributes	Position	96		
	Label	B_Sis3 = Avveie når de bør stoppes fra noe de forsøker å gjøre?		
	Type	Numeric		
	Format	F8		
	Measurement	Ordinal		
	Role	Input		
Valid Values	1	To a small degree	2	0,5%
	2	-	9	2,4%
	3	-	42	11,4%
	4	-	94	25,4%
	5	-	77	20,8%
	6	To a great degree	12	3,2%
Missing Values	-9999		134	36,2%

BSis4

		Value	Count	Percent
Standard Attributes	Position	97		
	Label	B_Sis4 = Avveie når de bør utfordres til å		

		gjøre noe de motsetter seg?		
	Type	Numeric		
	Format	F8		
	Measurement	Ordinal		
	Role	Input		
Valid Values	1	To a small degree	2	0,5%
	2	-	8	2,2%
	3	-	46	12,4%
	4	-	109	29,5%
	5	-	58	15,7%
	6	To a great degree	14	3,8%
Missing Values	-9999		133	35,9%

BSis5

		Value	Count	Percent
Standard Attributes	Position	98		
	Label	B_Sis5 = Møte dem hvis de blir verbalt utagerende?		
	Type	Numeric		
	Format	F8		
	Measurement	Ordinal		
	Role	Input		
Valid Values	1	To a small degree	5	1,4%
	2	-	10	2,7%
	3	-	26	7,0%
	4	-	77	20,8%
	5	-	84	22,7%
	6	To a great degree	35	9,5%
Missing Values	-9999		133	35,9%

BSis6

		Value	Count	Percent
Standard Attributes	Position	99		
	Label	B_Sis6 = Møte dem hvis de blir fysisk utagerende?		
	Type	Numeric		
	Format	F8		
	Measurement	Ordinal		
	Role	Input		
Valid Values	1	To a small degree	10	2,7%
	2	-	19	5,1%
	3	-	47	12,7%
	4	-	63	17,0%
	5	-	65	17,6%
	6	To a great degree	33	8,9%
Missing Values	-9999		133	35,9%

BSis7

		Value	Count	Percent
Standard Attributes	Position	100		

	Label	B_Sis7 = Regulere ditt eget stressnivå når du føler deg utfordret?		
	Type	Numeric		
	Format	F8		
	Measurement	Ordinal		
	Role	Input		
Valid Values	1	To a small degree	1	0,3%
	2	-	15	4,1%
	3	-	30	8,1%
	4	-	81	21,9%
	5	-	81	21,9%
	6	To a great degree	29	7,8%
Missing Values	-9999		133	35,9%

BUsc1

		Value	Count	Percent
Standard Attributes	Position	101		
	Label	B_Usc1 = Vi har en felles forståelse for hvordan vi bør møte dem		
	Type	Numeric		
	Format	F8		
	Measurement	Ordinal		
	Role	Input		
Valid Values	1	To a small degree	7	1,9%
	2	-	30	8,1%
	3	-	57	15,4%
	4	-	76	20,5%
	5	-	53	14,3%
	6	To a great degree	14	3,8%
Missing Values	-9999		133	35,9%

BUsc2

		Value	Count	Percent
Standard Attributes	Position	102		
	Label	B_Usc2 = Vi er bevisst på hvordan vi omtaler dem oss imellom		
	Type	Numeric		
	Format	F8		
	Measurement	Ordinal		
	Role	Input		
Valid Values	1	To a small degree	7	1,9%
	2	-	33	8,9%
	3	-	60	16,2%
	4	-	65	17,6%
	5	-	52	14,1%
	6	To a great degree	20	5,4%
Missing Values	-9999		133	35,9%

BUsc3

		Value	Count	Percent
Standard Attributes	Position	103		
	Label	B_Usc3 = Vi undersøker om de lever med pågående belastninger		
	Type	Numeric		
	Format	F8		
	Measurement	Ordinal		
	Role	Input		
Valid Values	1	To a small degree	1	0,3%
	2	-	29	7,8%
	3	-	49	13,2%
	4	-	67	18,1%
	5	-	63	17,0%
	6	To a great degree	28	7,6%
Missing Values	-9999		133	35,9%

BUsc4

		Value	Count	Percent
Standard Attributes	Position	104		
	Label	B_Usc4 = Vi hjelper hverandre til å se deres fremtidsmulighet er		
	Type	Numeric		
	Format	F8		
	Measurement	Ordinal		
	Role	Input		
Valid Values	1	To a small degree	1	0,3%
	2	-	22	5,9%
	3	-	40	10,8%
	4	-	77	20,8%
	5	-	76	20,5%
	6	To a great degree	21	5,7%
Missing Values	-9999		133	35,9%

BUsc5

		Value	Count	Percent
Standard Attributes	Position	105		
	Label	B_Usc5 = Vi jobber for å forebygge at de utsetter seg for risiko		
	Type	Numeric		
	Format	F8		
	Measurement	Ordinal		
	Role	Input		
Valid Values	1	To a small degree	2	0,5%
	2	-	13	3,5%

	3	-	31	8,4%
	4	-	63	17,0%
	5	-	81	21,9%
	6	To a great degree	47	12,7%
Missing Values	-9999		133	35,9%

BUsc6

		Value	Count	Percent
Standard Attributes	Position	106		
	Label	B_Usc6 = Vi har rutiner som gir dem trygghet og forutsigbarhet		
	Type	Numeric		
	Format	F8		
	Measurement	Ordinal		
	Role	Input		
Valid Values	1	To a small degree	4	1,1%
	2	-	12	3,2%
	3	-	32	8,6%
	4	-	58	15,7%
	5	-	75	20,3%
	6	To a great degree	56	15,1%
Missing Values	-9999		133	35,9%

BCsc1

		Value	Count	Percent
Standard Attributes	Position	107		
	Label	B_Csc1 = Vi samarbeider med andre tjenester om tiltakene		
	Type	Numeric		
	Format	F8		
	Measurement	Ordinal		
	Role	Input		
Valid Values	1	To a small degree	5	1,4%
	2	-	9	2,4%
	3	-	42	11,4%
	4	-	66	17,8%
	5	-	76	20,5%
	6	To a great degree	38	10,3%
Missing Values	-9999		134	36,2%

BCsc2

		Value	Count	Percent
Standard Attributes	Position	108		
	Label	B_Csc2 = Vi snakker om hvordan vi selv påvirker samspillet		
	Type	Numeric		
	Format	F8		
	Measurement	Ordinal		
	Role	Input		

Valid Values	1	To a small degree	7	1,9%
	2	-	39	10,5%
	3	-	54	14,6%
	4	-	63	17,0%
	5	-	49	13,2%
	6	To a great degree	24	6,5%
Missing Values	-9999		134	36,2%

BCsc3

		Value	Count	Percent
Standard Attributes	Position	109		
	Label	B_Csc3 = Vi har åpenhet om situasjoner vi syns er vanskelige		
	Type	Numeric		
	Format	F8		
	Measurement	Ordinal		
	Role	Input		
Valid Values	1	To a small degree	1	0,3%
	2	-	8	2,2%
	3	-	26	7,0%
	4	-	54	14,6%
	5	-	82	22,2%
	6	To a great degree	64	17,3%
Missing Values	-9999		135	36,5%

BCsc4

		Value	Count	Percent
Standard Attributes	Position	110		
	Label	B_Csc4 = Vi fremhever viktigheten av å ha traumekunnskap		
	Type	Numeric		
	Format	F8		
	Measurement	Ordinal		
	Role	Input		
Valid Values	1	To a small degree	24	6,5%
	2	-	42	11,4%
	3	-	57	15,4%
	4	-	50	13,5%
	5	-	42	11,4%
	6	To a great degree	21	5,7%
Missing Values	-9999		134	36,2%

BCsc5

		Value	Count	Percent
Standard Attributes	Position	111		
	Label	B_Csc5 = Vi er opptatt av å jobbe kultursensitivt		
	Type	Numeric		

	Format	F8		
	Measurement	Ordinal		
	Role	Input		
Valid Values	1	To a small degree	22	5,9%
	2	-	41	11,1%
	3	-	41	11,1%
	4	-	78	21,1%
	5	-	45	12,2%
	6	To a great degree	8	2,2%
Missing Values	-9999		135	36,5%

BCsc6

		Value	Count	Percent
Standard Attributes	Position	112		
	Label	B_Csc6 = Vi får veiledning på arbeid med utfordrende saker		
	Type	Numeric		
	Format	F8		
	Measurement	Ordinal		
	Role	Input		
Valid Values	1	To a small degree	27	7,3%
	2	-	36	9,7%
	3	-	41	11,1%
	4	-	46	12,4%
	5	-	58	15,7%
	6	To a great degree	27	7,3%
Missing Values	-9999		135	36,5%

TargGrup

		Value	Count	Percent
Standard Attributes	Position	113		
	Label	Target Pop = I hovedsak, hvilken målgruppe er jobben din rettet mot?		
	Type	Numeric		
	Format	F8		
	Measurement	Nominal		
	Role	Input		
Valid Values	1	Barn/ungdom	33	8,9%
	2	Voksne	232	62,7%
	3	Både barn/ungdom og voksne	44	11,9%
Missing Values	-9999		61	16,5%

Contact

		Value	Count	Percent
Standard Attributes	Position	114		
	Label	Contact Form = I hovedsak, hvordan er		

		arbeidet ditt relatert til dem som har sosiale og emosjonelle vansker?		
	Type	Numeric		
	Format	F8		
	Measurement	Nominal		
	Role	Input		
Valid Values	1	Eksklusivt brukere med vansker	99	26,8%
	2	Generell målgruppe	200	54,1%
	3	Kompetanseutvikling for fagpersoner	2	0,5%
	4	Administrasjon/ledelse-funksjon	4	1,1%
	5	Mer indirekte relasjon	7	1,9%
Missing Values	-9999		58	15,7%

Seniorit

		Value	Count	Percent
Standard Attributes	Position	115		
	Label	Seniority = Hvor lang erfaring har du med den typen arbeid?		
	Type	Numeric		
	Format	F8		
	Measurement	Scale		
	Role	Input		
N	Valid	306		
	Missing	64		
Central Tendency and Dispersion	Mean	3,71		
	Standard Deviation	2,080		
	Percentile 25	2,00		
	Percentile 50	3,00		
	Percentile 75	4,00		
Labeled Values	1	Mindre enn ett år	24	6,5%
	2	1-2 år	55	14,9%
	3	3-4 år	101	27,3%
	4	5-6 år	54	14,6%
	5	7-8 år	27	7,3%
	6	9-10 år	16	4,3%
	7	11-12 år	11	3,0%
	8	13-14 år	4	1,1%
	9	15-16 år	9	2,4%
	10	17-18 år	1	0,3%
	11	19-20 år	1	0,3%
	12	21-22 år	1	0,3%
	13	23-24 år	1	0,3%
	14	25-26 år	1	0,3%
	15	27-28 år	0	0,0%
	16	29-30 år	0	0,0%
	17	Mer enn 30 år	0	0,0%

Edu

		Value	Count	Percent
Standard Attributes	Position	116		
	Label	Basic Education = Hvilken type grunnutdanning har du?		
	Type	Numeric		
	Format	F8		
	Measurement	Ordinal		
	Role	Input		
Valid Values	1	Grunnskole	0	0,0%
	2	Videregående skole	5	1,4%
	3	Fagskole el. tilsvarende	0	0,0%
	4	Profesjonsutd., 3-4 årig	291	78,6%
	5	Profesjonsutd., 5 år el. mer	13	3,5%
	6	Annen type utdanning	3	0,8%
Missing Values	-9999		58	15,7%

PrioComp

		Value	Count	Percent
Standard Attributes	Position	117		
	Label	Prior Trauma Training = Etter din grunnutdanning, hvor mye kompetanseutvikling om traumeforståelse og traumearbeid har du deltatt på?		
	Type	Numeric		
	Format	F8		
	Measurement	Ordinal		
	Role	Input		
Valid Values	1	Lite	138	37,3%
	2	Noe	110	29,7%
	3	En god del	38	10,3%
	4	En god del	16	4,3%
	5	Mye	9	2,4%
	6	Svært mye	0	0,0%
Missing Values	-9999		59	15,9%

Service

		Value	Count	Percent
Standard Attributes	Position	118		
	Label	Service affiliation = I hvilken type tjeneste jobber du?		
	Type	Numeric		
	Format	F8		
	Measurement	Nominal		

	Role	Input		
Valid Values	1	Barnehage	0	0,0%
	2	Barnevern, kommunalt	8	2,2%
	3	Barnevern, privat	8	2,2%
	4	Barnevern, statlig	4	1,1%
	5	Brukerorganisasjon	0	0,0%
	6	Familievern	0	0,0%
	7	Flyktning-/asyltjeneste	1	0,3%
	8	Forsvaret	0	0,0%
	9	Frivillig organisasjon	1	0,3%
	10	Kommunal helsetjeneste	68	18,4%
	11	Kommunal admin./ledelse	1	0,3%
	12	Kommunal omsorgs-/sosialtjeneste	34	9,2%
	13	Kunnskaps-/kompetansesenter	0	0,0%
	14	Kriminalomsorg	1	0,3%
	15	NAV	8	2,2%
	16	Politi	1	0,3%
	17	PPT	0	0,0%
	18	Skole, barn/ungdom	1	0,3%
	19	Skole, Videregående	1	0,3%
	20	Spesialisthelsetjeneste	151	40,8%
	21	Trossamfunn/kirke	0	0,0%
	22	Universitet/høyskole, ansatt	0	0,0%
	23	Universitet/høyskole, student	0	0,0%
	24	Annen tjeneste	22	5,9%
Missing Values	-9999		60	16,2%

DIContfrm

		Value	Count	Percent
Standard Attributes	Position	137		
	Label	gDIC cont form: 1=Jobber ekslusivt med dem som har utfordringer, 2=møter dem i generelle tjenester. NB: Denne blir kalt «ROLE TYPE» teksten metodedelen i artikkelen, og står som «Target		

		group specificity» i tabellene. Bør endres til «Role type» alle stede		
	Type	Numeric		
	Format	F8		
	Measurement	Ordinal		
	Role	Input		
Valid Values	1	Jobber ekslusivt med dem som sliter	99	26,8%
	2	Møter de som sliter i generelle tjenester	200	54,1%
Missing Values	-9999		71	19,2%

DICTarg

		Value	Count	Percent
Standard Attributes	Position	138		
	Label	DIC targ: 1=Jobber med barn/unge eller både barn/unge og voksne, 2=jobber kun med voksne. NB: Denne blir kalt «TARGET AGE GROUP» i tekst i metodedelen i artikkelen, og «Age group served» i tabeller. Bør endres til «Target age group» alle steder.		
	Type	Numeric		
	Format	F8		
	Measurement	Ordinal		
	Role	Input		
Valid Values	1	Jobber med barn/unge eller både barn/unge og voksne	77	20,8%
	2	Jobber kun med voksne	232	62,7%
Missing Values	-9999		61	16,5%

EduRec

		Value	Count	Percent
Standard Attributes	Position	139		
	Label	RECO_Educat		
	Type	Numeric		
	Format	F8		
	Measurement	Ordinal		
	Role	Input		
Valid Values	1	Grunnskole	0	0,0%
	2	Videregående	5	1,4%

		skole		
	3	Fagskole	0	0,0%
	4	Universitet/høyskole, 3-4 år	291	78,6%
	5	Universitet/høyskole, 5+ år	13	3,5%
Missing Values	-9999		61	16,5%

SerAffDi

		Value	Count	Percent
Standard Attributes	Position	140		
	Label	DICO serv aff: 1: 1=jobber i kommunale/allemenne tjenester, 2=jobber i spesislisthelsetjeneste. Denne heter «WORKPLACE TYPE » i tekst i metodedelen av artikkelen, og «Workplace affiliation» i tabeller. Bør endres til «Workplace type» alle steder.		
	Type	Numeric		
	Format	F8		
	Measurement	Ordinal		
	Role	Input		
Valid Values	1	Jobber i kommunale/allmenne tjenester	159	43,0%
	2	Jobber i spesialisthelsetjeneste	151	40,8%
Missing Values	-9999		60	16,2%

PhyDigDi

		Value	Count	Percent
Standard Attributes	Position	141		
	Label	DICO_Phys_dig		
	Type	Numeric		
	Format	F8		
	Measurement	Ordinal		
	Role	Input		
Valid Values	1	Fysisk eller blandet deltakelse	146	39,5%
	2	Kun digital deltakelse	83	22,4%
Missing Values	-9999		141	38,1%

SUMCont

		Value	Count	Percent
Standard Attributes	Position	142		

	Label	SUM_Content = Sumskåre for ulike innhold i programmene		
	Type	Numeric		
	Format	F8		
	Measurement	Ordinal		
	Role	Input		
Valid Values	0		137	37,0%
	1		84	22,7%
	2		69	18,6%
	3		57	15,4%
	4		19	5,1%
	5		3	0,8%
	6		1	0,3%

ARDYSUM

		Value
Standard Attributes	Position	143
	Label	A_RDY_SUM = Sumscore for all items under the domain Readyness_PRE
	Type	Numeric
	Format	F8
	Measurement	Scale
	Role	Input
N	Valid	315
	Missing	55
Central Tendency and Dispersion	Mean	15,32
	Standard Deviation	2,810
	Percentile 25	14,00
	Percentile 50	16,00
	Percentile 75	18,00

AAGNSUM

		Value
Standard Attributes	Position	144
	Label	A_AGN_SUM = Sumscore for all items under the domain Agency_PRE
	Type	Numeric
	Format	F8
	Measurement	Scale
	Role	Input
N	Valid	315
	Missing	55
Central Tendency and Dispersion	Mean	21,44
	Standard Deviation	3,909
	Percentile 25	19,00
	Percentile 50	21,00
	Percentile 75	24,00

ARFXSUM

		Value
Standard Attributes	Position	145

	Label	A_RFX_SUM = Sumscore for all items under the domain Reflexivity_PRE
	Type	Numeric
	Format	F8
	Measurement	Scale
	Role	Input
N	Valid	310
	Missing	60
Central Tendency and Dispersion	Mean	25,75
	Standard Deviation	5,052
	Percentile 25	23,00
	Percentile 50	26,00
	Percentile 75	29,00

ATKWSUM

		Value
Standard Attributes	Position	146
	Label	A_TKW_SUM = Sumscore for all items under the domain Thematic Knowledge_PRE
	Type	Numeric
	Format	F8
	Measurement	Scale
	Role	Input
N	Valid	313
	Missing	57
Central Tendency and Dispersion	Mean	26,57
	Standard Deviation	8,763
	Percentile 25	20,00
	Percentile 50	26,00
	Percentile 75	33,00

ACKWSUM

		Value
Standard Attributes	Position	147
	Label	A_CKW_SUM = Sumscore for all items under the domain Conseptual Knowledge_PRE
	Type	Numeric
	Format	F8
	Measurement	Scale
	Role	Input
N	Valid	311
	Missing	59
Central Tendency and Dispersion	Mean	21,45
	Standard Deviation	6,807
	Percentile 25	17,00
	Percentile 50	22,00
	Percentile 75	26,00

AKNOWSUM

		Value
Standard Attributes	Position	148
	Label	A_KNOWLEDE_SUM = Sumscore for both domains Knowledge_PRE
	Type	Numeric
	Format	F8
	Measurement	Scale
	Role	Input
N	Valid	310
	Missing	60
Central Tendency and Dispersion	Mean	48,00
	Standard Deviation	14,575
	Percentile 25	38,00
	Percentile 50	48,50
	Percentile 75	59,00

ASUSSUM

		Value
Standard Attributes	Position	149
	Label	A_SUS_SUM = Sumscore for all items under the domain Supportive Skills_PRE
	Type	Numeric
	Format	F8
	Measurement	Scale
	Role	Input
N	Valid	312
	Missing	58
Central Tendency and Dispersion	Mean	25,30
	Standard Deviation	7,909
	Percentile 25	20,00
	Percentile 50	25,00
	Percentile 75	31,00

ASISSUM

		Value
Standard Attributes	Position	150
	Label	A_SIS_SUM = Sumscore for all items under the domain Situational Skills_PRE
	Type	Numeric
	Format	F8
	Measurement	Scale
	Role	Input
N	Valid	313
	Missing	57
Central Tendency and Dispersion	Mean	24,73
	Standard Deviation	7,251
	Percentile 25	20,00
	Percentile 50	25,00
	Percentile 75	29,00

ASKILSUM

		Value
Standard Attributes	Position	151
	Label	A_SKILLS_SUM = Sumscore for both domains Skills_PRE
	Type	Numeric
	Format	F8
	Measurement	Scale
	Role	Input
N	Valid	311
	Missing	59
Central Tendency and Dispersion	Mean	50,08
	Standard Deviation	14,102
	Percentile 25	40,00
	Percentile 50	51,00
	Percentile 75	60,00

AUSCSUM

		Value
Standard Attributes	Position	152
	Label	COMPUTE A_USC_SUM=A_Usc1 + A_Usc2 + A_Usc3 + A_Usc4 + A_Usc5 + A_Usc6
	Type	Numeric
	Format	F8
	Measurement	Scale
	Role	Input
N	Valid	312
	Missing	58
Central Tendency and Dispersion	Mean	23,38
	Standard Deviation	6,156
	Percentile 25	20,00
	Percentile 50	24,00
	Percentile 75	28,00

ACSCSUM

		Value
Standard Attributes	Position	153
	Label	A_CSC_SUM = Sumscore for all items under the domain Collegue Supportive Culture_PRE
	Type	Numeric
	Format	F8
	Measurement	Scale
	Role	Input
N	Valid	307
	Missing	63
Central Tendency and Dispersion	Mean	22,51
	Standard Deviation	6,363

	Percentile 25	18,00
	Percentile 50	23,00
	Percentile 75	27,00

ACULTSUM

		Value
Standard Attributes	Position	154
	Label	A_CULTURE_SUM = Sumscore for both domains Culture_PRE
	Type	Numeric
	Format	F8
	Measurement	Scale
	Role	Input
N	Valid	306
	Missing	64
Central Tendency and Dispersion	Mean	45,90
	Standard Deviation	11,768
	Percentile 25	39,00
	Percentile 50	46,00
	Percentile 75	55,00

BRDYSUM

		Value
Standard Attributes	Position	155
	Label	B_RDY_SUM = Sumscore for all items under the domain Readyness_POST
	Type	Numeric
	Format	F8
	Measurement	Scale
	Role	Input
N	Valid	238
	Missing	132
Central Tendency and Dispersion	Mean	15,88
	Standard Deviation	2,510
	Percentile 25	15,00
	Percentile 50	17,00
	Percentile 75	18,00

BAGNSUM

		Value
Standard Attributes	Position	156
	Label	B_AGN_SUM = Sumscore for all items under the domain Agency_POST
	Type	Numeric
	Format	F8
	Measurement	Scale
	Role	Input
N	Valid	237
	Missing	133
Central Tendency and	Mean	22,46

Dispersion	Standard Deviation	3,634
	Percentile 25	20,00
	Percentile 50	23,00
	Percentile 75	25,00

BRFXSUM

		Value
Standard Attributes	Position	157
	Label	B_RFX_SUM = Sumscore for all items under the domain ReflexivitY_POST
	Type	Numeric
	Format	F8
	Measurement	Scale
	Role	Input
N	Valid	235
	Missing	135
Central Tendency and Dispersion	Mean	27,70
	Standard Deviation	4,600
	Percentile 25	25,00
	Percentile 50	28,00
	Percentile 75	31,00

BTKWSUM

		Value
Standard Attributes	Position	158
	Label	B_TKW_SUM = Sumscore for all items under the domain Thematic Knowledge_POST
	Type	Numeric
	Format	F8
	Measurement	Scale
	Role	Input
N	Valid	234
	Missing	136
Central Tendency and Dispersion	Mean	36,72
	Standard Deviation	5,340
	Percentile 25	34,00
	Percentile 50	37,00
	Percentile 75	40,00

BCKWSUM

		Value
Standard Attributes	Position	159
	Label	B_CKW_SUM = Sumscore for all items under the domain Conseptual Knowledge_POST
	Type	Numeric
	Format	F8

	Measurement	Scale
	Role	Input
N	Valid	237
	Missing	133
Central Tendency and Dispersion	Mean	27,99
	Standard Deviation	4,305
	Percentile 25	25,00
	Percentile 50	28,00
	Percentile 75	30,00

BKNOWSUM

		Value
Standard Attributes	Position	160
	Label	B_KNOWLEDE_SUM = Sumscore for both domains Knowledge_POST
	Type	Numeric
	Format	F8
	Measurement	Scale
	Role	Input
N	Valid	233
	Missing	137
Central Tendency and Dispersion	Mean	64,77
	Standard Deviation	8,866
	Percentile 25	60,00
	Percentile 50	65,00
	Percentile 75	70,00

BSUSSUM

		Value
Standard Attributes	Position	161
	Label	B_SUS_SUM = Sumscore for all items under the domain Supportive Skills_POST
	Type	Numeric
	Format	F8
	Measurement	Scale
	Role	Input
N	Valid	234
	Missing	136
Central Tendency and Dispersion	Mean	32,91
	Standard Deviation	5,973
	Percentile 25	30,00
	Percentile 50	33,00
	Percentile 75	37,00

BSISSUM

		Value
Standard Attributes	Position	162
	Label	B_SIS_SUM = Sumscore for all items under the domain Situational

		Skills_POST
	Type	Numeric
	Format	F8
	Measurement	Scale
	Role	Input
N	Valid	235
	Missing	135
Central Tendency and Dispersion	Mean	29,05
	Standard Deviation	5,896
	Percentile 25	26,00
	Percentile 50	30,00
	Percentile 75	33,00

BSKILSUM

		Value
Standard Attributes	Position	163
	Label	B_SKILLS_SUM = Sumscore for both domains Skills_POST
	Type	Numeric
	Format	F8
	Measurement	Scale
	Role	Input
N	Valid	232
	Missing	138
Central Tendency and Dispersion	Mean	61,93
	Standard Deviation	10,939
	Percentile 25	55,00
	Percentile 50	63,00
	Percentile 75	70,00

BUSCSUM

		Value
Standard Attributes	Position	164
	Label	B_USC_SUM = Sumscore for all items under the domain User Supportive Culture_POST
	Type	Numeric
	Format	F8
	Measurement	Scale
	Role	Input
N	Valid	237
	Missing	133
Central Tendency and Dispersion	Mean	24,67
	Standard Deviation	5,877
	Percentile 25	20,00
	Percentile 50	25,00
	Percentile 75	29,00

BCSCSUM

		Value
Standard Attributes	Position	165
	Label	B_CSC_SUM = Sumscore for all items under the

		domain Colleague Supportive Culture_POST
	Type	Numeric
	Format	F8
	Measurement	Scale
	Role	Input
N	Valid	233
	Missing	137
Central Tendency and Dispersion	Mean	23,39
	Standard Deviation	5,926
	Percentile 25	19,00
	Percentile 50	24,00
	Percentile 75	28,00

BCULTSUM

		Value
Standard Attributes	Position	166
	Label	B_CULTURE_SUM = Sumscore for both domains Culture_POST
	Type	Numeric
	Format	F8
	Measurement	Scale
	Role	Input
N	Valid	233
	Missing	137
Central Tendency and Dispersion	Mean	48,09
	Standard Deviation	10,963
	Percentile 25	40,00
	Percentile 50	49,00
	Percentile 75	55,00

ATOTAL

		Value
Standard Attributes	Position	167
	Label	A_TOTAL = Sum score for all TANDEM test items_PRE
	Type	Numeric
	Format	F8
	Measurement	Scale
	Role	Input
N	Valid	295
	Missing	75
Central Tendency and Dispersion	Mean	206,46
	Standard Deviation	38,787
	Percentile 25	181,00
	Percentile 50	206,00
	Percentile 75	236,00

BTOTAL

		Value
Standard Attributes	Position	168
	Label	B_TOTAL = Sum score for all

		TANDEM test items_POST
	Type	Numeric
	Format	F8
	Measurement	Scale
	Role	Input
N	Valid	214
	Missing	156
Central Tendency and Dispersion	Mean	241,50
	Standard Deviation	27,932
	Percentile 25	226,00
	Percentile 50	241,00
	Percentile 75	262,00

DIFFRDY

		Value
Standard Attributes	Position	169
	Label	DIFF_RDY = Differansen mellom pre og post sumskårene for READY
	Type	Numeric
	Format	F8
	Measurement	Scale
	Role	Input
N	Valid	185
	Missing	185
Central Tendency and Dispersion	Mean	,38
	Standard Deviation	2,828
	Percentile 25	-1,00
	Percentile 50	,00
	Percentile 75	2,00

DIFFAGN

		Value
Standard Attributes	Position	170
	Label	DIFF_AGN = Differansen mellom pre og post sumskårene for AGENCY
	Type	Numeric
	Format	F8
	Measurement	Scale
	Role	Input
N	Valid	186
	Missing	184
Central Tendency and Dispersion	Mean	1,48
	Standard Deviation	3,392
	Percentile 25	-1,00
	Percentile 50	1,00
	Percentile 75	4,00

DIFFRFX

		Value
Standard Attributes	Position	171
	Label	DIFF_RFX = Differansen

		mellom pre og post sumskårene for REFLEXIVITY
	Type	Numeric
	Format	F8
	Measurement	Scale
	Role	Input
N	Valid	180
	Missing	190
Central Tendency and Dispersion	Mean	2,51
	Standard Deviation	5,133
	Percentile 25	-1,00
	Percentile 50	2,00
	Percentile 75	6,00

DIFFTKW

		Value
Standard Attributes	Position	172
	Label	DIFF_TKW = Differansen mellom pre og post sumskårene for THEMATIC KNOWLEDGE
	Type	Numeric
	Format	F8
	Measurement	Scale
	Role	Input
N	Valid	181
	Missing	189
Central Tendency and Dispersion	Mean	10,96
	Standard Deviation	8,055
	Percentile 25	5,00
	Percentile 50	11,00
	Percentile 75	16,00

DIFFCKW

		Value
Standard Attributes	Position	173
	Label	DIFF_CKW = Differansen mellom pre og post sumskårene for CONCEPTUAL KNOWLEDGE
	Type	Numeric
	Format	F8
	Measurement	Scale
	Role	Input
N	Valid	182
	Missing	188
Central Tendency and Dispersion	Mean	6,96
	Standard Deviation	5,889
	Percentile 25	2,00
	Percentile 50	6,00
	Percentile 75	11,00

DIFFKNOW

		Value
Standard Attributes	Position	174
	Label	DIFF_KNOWLEDGE = Differansen mellom pre og post sumskårene for KNOWLEDGE TOTALT
	Type	Numeric
	Format	F8
	Measurement	Scale
	Role	Input
N	Valid	178
	Missing	192
Central Tendency and Dispersion	Mean	18,01
	Standard Deviation	12,908
	Percentile 25	9,00
	Percentile 50	18,00
	Percentile 75	27,00

DIFFSUS

		Value
Standard Attributes	Position	175
	Label	DIFF_SUS = Differansen mellom pre og post sumskårene for SUPPORTIVE SKILLS
	Type	Numeric
	Format	F8
	Measurement	Scale
	Role	Input
N	Valid	183
	Missing	187
Central Tendency and Dispersion	Mean	8,64
	Standard Deviation	6,840
	Percentile 25	4,00
	Percentile 50	8,00
	Percentile 75	13,00

DIFFSIS

		Value
Standard Attributes	Position	176
	Label	DIFF_SIS = Differansen mellom pre og post sumskårene for SITUATIONAL SKILLS
	Type	Numeric
	Format	F8
	Measurement	Scale
	Role	Input
N	Valid	182
	Missing	188
Central Tendency and Dispersion	Mean	4,97
	Standard Deviation	5,422

	Percentile 25	1,00
	Percentile 50	5,00
	Percentile 75	9,00

DIFFSKIL

		Value
Standard Attributes	Position	177
	Label	DIFF_SKILLS = Differansen mellom pre og post sumskårene for SKILLS TOTALT
	Type	Numeric
	Format	F8
	Measurement	Scale
	Role	Input
N	Valid	181
	Missing	189
Central Tendency and Dispersion	Mean	13,67
	Standard Deviation	11,194
	Percentile 25	6,00
	Percentile 50	13,00
	Percentile 75	23,00

DIFFUSC

		Value
Standard Attributes	Position	178
	Label	DIFF_USC = Differansen mellom pre og post sumskårene for USER SUPPORTIVE CULTURE
	Type	Numeric
	Format	F8
	Measurement	Scale
	Role	Input
N	Valid	183
	Missing	187
Central Tendency and Dispersion	Mean	1,96
	Standard Deviation	4,777
	Percentile 25	-1,00
	Percentile 50	2,00
	Percentile 75	5,00

DIFFCSC

		Value
Standard Attributes	Position	179
	Label	DIFF_CSC = Differansen mellom pre og post sumskårene for COLLEAGUE SUPPORTIVE CULTURE
	Type	Numeric
	Format	F8

	Measurement	Scale
	Role	Input
N	Valid	180
	Missing	190
Central Tendency and Dispersion	Mean	1,84
	Standard Deviation	4,442
	Percentile 25	-1,00
	Percentile 50	2,00
	Percentile 75	4,00

DIFFCULT

		Value
Standard Attributes	Position	180
	Label	DIFF_CULTURE = Differansen mellom pre og post sumskårene for CULTURE TOTALT
	Type	Numeric
	Format	F8
	Measurement	Scale
	Role	Input
N	Valid	180
	Missing	190
Central Tendency and Dispersion	Mean	3,83
	Standard Deviation	8,083
	Percentile 25	-1,00
	Percentile 50	4,00
	Percentile 75	9,00

DIFTOTAL

		Value
Standard Attributes	Position	181
	Label	DIFF_TOTAL = Differansen mellom pre og post sumskårene for TOTALSKÅREN E for hele TANDEM
	Type	Numeric
	Format	F8
	Measurement	Scale
	Role	Input
N	Valid	162
	Missing	208
Central Tendency and Dispersion	Mean	40,24
	Standard Deviation	29,854
	Percentile 25	20,00
	Percentile 50	39,00
	Percentile 75	59,00

WplProp

		Value	Count	Percent
Standard Attributes	Position	182		
	Label	Treedeling av andel fra		

		arbeidsplassen som deltok		
	Type	Numeric		
	Format	F8		
	Measurement	Nominal		
	Role	Input		
Valid Values	1		175	47,3%
	2		4	1,1%
	3		21	5,7%
Missing Values	-9999		170	45,9%

MD_A

		Value	Count	Percent
Standard Attributes	Position	183		
	Label	<none>		
	Type	Numeric		
	Format	F8		
	Measurement	Nominal		
	Role	Input		
Valid Values	0		295	79,7%
	1		13	3,5%
	2		1	0,3%
	3		1	0,3%
	4		1	0,3%
	6		2	0,5%
	9		1	0,3%
	47		2	0,5%
	52		1	0,3%
	55		53	14,3%

MD_B

		Value	Count	Percent
Standard Attributes	Position	184		
	Label	<none>		
	Type	Numeric		
	Format	F8		
	Measurement	Nominal		
	Role	Input		
Valid Values	0		214	57,8%
	1		20	5,4%
	4		1	0,3%
	6		1	0,3%
	12		1	0,3%
	27		2	0,5%
	47		2	0,5%
	52		1	0,3%
	55		128	34,6%

Data_A

		Value	Count	Percent
Standard Attributes	Position	185		
	Label	<none>		
	Type	Numeric		
	Format	F8		
	Measurement	Nominal		
	Role	Input		
Valid Values	0	MD	53	14,3%

	1	Data	317	85,7%

Data_B

		Value	Count	Percent
Standard Attributes	Position	186		
	Label	<none>		
	Type	Numeric		
	Format	F8		
	Measurement	Nominal		
	Role	Input		
Valid Values	0	MD	128	34,6%
	1	Data	242	65,4%

StudData

		Value	Count	Percent
Standard Attributes	Position	187		
	Label	<none>		
	Type	Numeric		
	Format	F8		
	Measurement	Nominal		
	Role	Input		
Valid Values	1	Not much	128	34,6%
	2		53	14,3%
	3		189	51,1%
	6	Very much	0	0,0%
