# Supplementary material for: The sensitivity of TANDEM – A new measure of trauma competence
Source: PLoS One. 2026 Jan 28;21(1):e0339858. doi: 10.1371/journal.pone.0339858 (PMC12851466; doi:10.1371/journal.pone.0339858)
Supplement: Attachment — Submitted filename: The sensitivity of TANDEM.pdf [file pone.0339858.s007.pdf]

The authors have put in substantial work across these revisions. They clarify the sampling denominator and now compute the response rate against the full three-cohort denominator (n=339), arriving at 167 paired responses and a final analytic sample of 163 after exclusions—an overall response rate of 48.1%. They also note that only about three-quarters of enrolled students attended lectures, which is relevant context for interpreting that rate. At the same time, the tracked-changes manuscript still contains legacy text reporting “74% … adjusted to 70%,” creating a direct contradiction that needs to be resolved before acceptance. Only the updated calculation (339 denominator; 48.1%) should remain.

The measurement and analysis strategy has advanced meaningfully. The authors now distinguish reflective domains (Readiness, Agency, Reflexivity) from the formative domains (Knowledge, Skills, Work/Organizational Culture), test longitudinal measurement invariance via CFA for the reflective domains, and estimate change using two-time-point latent growth–equivalent models with FIML. This is a substantial step up in rigor.

The limitations discussion has also expanded. They explicitly caution about the correlation between baseline and change (i.e., regression to the mean), cite relevant methodology, and frame the invariance checks in relation to possible response-shift phenomena—appropriate concerns in pre–post designs. They report that effect sizes were estimated within the LGC framework and refer to supplementary tables, which is appropriate, but several reporting details still need tightening in the main PDF.

These are genuine methodological improvements. The remaining issues are largely about internal consistency and transparent reporting.

On the sampling frame and response rate

The “Sample” section now reports the exact cohort sizes (121, 110, 108; total = 339), 167 paired responses, and a final N=163 after four exclusions, yielding a 48.1% overall response rate based on the full denominator. The paragraph also clarifies that only ~75% of students attended the lectures where TANDEM was administered. This is much clearer than before. However, other paragraphs in the tracked-changes manuscript still carry the older “74% … adjusted to 70%” language, which conflicts with the updated computation. Please delete the legacy text and keep only the paragraph with the 339 denominator and 48.1% rate.

On measurement models, invariance, and change modeling

This is where the authors have made the most progress. They now explicitly treat Readiness, Agency, and Reflexivity as reflective constructs, test configural/metric/scalar/strict invariance for these domains via longitudinal CFA, and then estimate change using constrained two-time-point LGC models with FIML (random-intercept, fixed-slope), which is aligned with current best practice.

Two points need attention:

1. **Legacy EFA text remains.** Despite the pivot to CFA and invariance testing, the R3 file still contains an EFA paragraph with SPSS output embedded in the methods/results. That text conflicts with the current CFA-based approach and should be removed or, if the authors believe it is informative, moved to a short, clearly labeled historical note (e.g., “Analytic evolution from EFA to CFA”).

2. **Ambiguous invariance phrasing.** In the invariance write-up, the text says constraining intercepts led to a “non-significant model fit” and a “statistically significant reduction in chi-square,” and then takes this as evidence for strong (scalar) invariance. Typically, adding constraints **increases** χ^2, and scalar invariance is supported when the χ^2 difference is **not** significant and/or when changes in incremental fit (e.g., $\Delta CFI \leq .01$) are small. Please clarify the direction and criteria used (e.g., ΔCFI, ΔRMSEA, χ^2-difference) and correct the “reduction in chi-square” wording if it is indeed backwards.

Relatedly, the text notes that “model fit was not relevant” for the restricted two-time-point LGC models. It would help readers to add one sentence explaining that—with the imposed constraints—these models become just-identified, which renders global fit indices uninformative.

On effect sizes, confidence intervals, and regression reporting

Within the LGC framework, the authors state they computed effect sizes by dividing estimated change by the pre-test SD, and they reference supplementary tables. Please ensure that:

- Pre–post effect sizes **with 95% CIs** are visible in the main PDF (not only in DOCX supplements), at least for the total scale and the six domains.

- Wherever regression/LGC coefficients are reported, it is clear whether these are **standardized or unstandardized**, and confidence intervals are provided in the main tables.

- Given the number of tests across six domains and several predictors, the results should be explicitly framed as **exploratory**, with a brief note on whether any multiplicity control (e.g., FDR) was considered.

On regression to the mean and response shift

The discussion now directly acknowledges that baseline–change correlations may reflect regression to the mean and references appropriate methodological sources. Response-shift considerations are discussed alongside the invariance checks. This is an improvement and appropriate for a pre–post study design.

On terminology and naming consistency

Terminology has improved (e.g., “course” vs “TCP”), but domain labeling still varies between **“Work culture”** and **“Organisational/Organizational culture”** across sections and tables. For example, the manuscript’s text refers to “Organisational Culture,” while the results narrative and tables sometimes say “Work culture.” Please standardize the domain label across abstract, methods, results, and tables. Also choose either UK (“organisational”) or US (“organizational”) spelling and apply it consistently throughout.

On data availability and reproducibility

The submission form now states that the dataset has been deposited in the institution’s repository and will be available without restriction, with the DOI “awaiting” issuance. This aligns with PLOS ONE policy; however, the **actual DOI/URL** must be inserted into the **Data Availability** statement before acceptance. A minimal **data dictionary/codebook** describing variable names, scales, and any recoding (including the dichotomizations below) would materially improve reproducibility.

On predictor handling and dichotomization

The methods indicate that several predictors were dichotomized (Target age group, Workplace type, Role type), with some role categories excluded to achieve a binary split. While pragmatic, dichotomization discards information and can reduce power. Where feasible, retain the original categories using dummy variables or ordered terms and consider sensitivity checks if sparse cells are a concern. At minimum, justify the necessity of dichotomization and report the **original** distributions (some already appear in Tables 1–2).

On figures and readability

A compact figure that shows domain‑level mean change with 95% CIs (e.g., a forest plot) would aid readers who prefer a visual summary over scanning dense tables. The current PDF routes key tables to a **DOCX** supplement; at least one figure in-paper would help readers grasp the pattern of results at a glance.

On authorship and metadata

Authorship/corresponding‑author information is inconsistent across the package: the Editorial Manager front matter lists **Dag Ø. Nordanger** as the corresponding author, while the tracked‑changes manuscript lists **Alina Coman**. Please reconcile author order and the corresponding author across both EM metadata and the manuscript file.

On minor editorial issues

Several tracked‑changes remnants remain and should be cleaned up. For example, there is at least one placeholder (“[INSERT TABLE 3 ABOUT HERE]”) still present in the R3 text. Please

remove all placeholders and ensure that final tables/figures are properly embedded per PLOS format. Also double-check decimal punctuation and spelling conventions for consistency.

Status of prior concerns

- **Cohort denominator/response rate:** Mostly resolved with clear reporting (n=339; 48.1%), but the contradictory "70%" text must be deleted.

- **Measurement model & change estimation:** Major improvement with CFA invariance and LGC/FIML. Invariance criteria and phrasing need clarification; remove or relocate legacy EFA text.

- **Effect sizes & CIs:** Claimed/estimated under LGC; ensure effect sizes and CIs are visible in the main PDF with clear labeling of standardized vs unstandardized coefficients, not only in supplements.

- **Regression-to-mean/response shift:** Now appropriately discussed.

- **Terminology:** "Work culture" vs "Organisational/Organizational culture" still inconsistent; harmonize.

- **Data availability:** Repository deposit indicated; DOI still pending—must be inserted. Provide a codebook.

- **Figures:** Still absent; adding a simple change-summary figure would help.

- **Authorship metadata:** Inconsistent between EM and manuscript—align before acceptance.

What I'm recommending to the authors

1. **Finalize the Sample section** by keeping only the updated paragraph with the 339 denominator, 167 paired responses, 4 exclusions, final N=163, and overall 48.1% response rate; remove the legacy "70%" language.

2. **Clarify invariance testing** by stating the exact decision rules (e.g., ΔCFI ≤ .01; ΔRMSEA ≤ .015; non-significant χ^2 difference), and correct the "reduction in χ^2" phrasing if reversed. Remove or clearly sequester the residual **EFA** paragraph.

3. **Make the analysis outputs visible in-paper.** Ensure pre–post effect sizes **with 95% CIs** and clearly labeled **standardized vs unstandardized** coefficients appear in the main tables, not only in DOCX supplements. Briefly acknowledge multiplicity and whether any FDR-style control was considered.

4. **Justify or reduce dichotomization** of Target age group, Workplace type, and Role type; if feasible, re-run with dummy/ordered terms and report sensitivity analyses.

5. **Harmonize terminology.** Pick either "Work culture" or "Organisational/Organizational culture" and use it consistently across text and tables; choose UK or US spelling and apply uniformly.

6. **Lock down data availability** by inserting the repository DOI/URL and adding a short codebook (variables, scales, and recodes).

7. **Align authorship metadata** so the author order and corresponding author in EM match the manuscript.

8. **Tidy presentation** by removing all placeholders/duplications and ensuring the final table/figure set conforms to PLOS formatting.
