# Supplementary material for: The sensitivity of TANDEM – A new measure of trauma competence
Source: PLoS One. 2026 Jan 28;21(1):e0339858. doi: 10.1371/journal.pone.0339858 (PMC12851466; doi:10.1371/journal.pone.0339858)
Supplement: Attachment — Submitted filename: Response to Reviewers.docx [file pone.0339858.s004.docx]

**Dear editor**

Thank you for the constructive and encouraging feedback on our manuscript “The sensitivity of TANDEM”. We appreciate the opportunity you gave to revise and resubmit it. You have summarized and organized the reviewers’ comments in the eight points below and invite us to relate our responses to these points. Under each point, we have included [in square brackets] the exact phrasing of the reviewer-comment it concerns. Please, for each point, see our description of how we have responded to the comments, including the text revisions they resulted in.

Please note that regarding points 5–8, which are all comments from Reviewer 2, it has already been clarified with the editor that these should be understood as suggestions for future studies that are needed before any final conclusions can be drawn about the psychometric properties of TANDEM. This means that, in the current study, these unresolved questions must be addressed in terms of caution and limitations with respect to the study’s conclusions. Our ways of solving it are outlined below.

1- *The study assesses self-perceived competence rather than actual performance.*
*Suggest comparing TANDEM scores with performance-based measures or service user perceptions of trauma-informed care for future validation.*
[This point is brought up by Reviewer 1: “The original study (referenced as 24) which established criterion validity, did so by comparing TANDEM scores to self-reported training/education (RMT). I think it might be worth considering that a comparison to performance as opposed to self-reported competence would be a useful future addition to the validation of TANDEM. Does TANDEM score align with service user’s perception of whether care is trauma informed?”]

Our response: Yes, we fully agree. Until now, both in the original study and in this one, validity and psychometric properties have been assessed by the means of variables, based on self-reports, integral to the instrument. The best assurance of criterion validity would be independent measures capturing the actual practice. As also several points below concern next steps in the instrument development and validation process, we have included a new subheading called “Limitations and next steps” in the Discussion section, where the point you make here is included. There, the part addressing this first point reads:

“Another uncertainty related to TANDEM as a competence measure concerns the instrument's correspondence with actual practical or demonstrated professional competence. In other words, to what extent do increases in scores reflect real improvements in the quality of services provided? At present, the only criteria used are those embedded within the instrument itself. Moving forward, testing TANDEM against performance-based measures, as well as criteria that capture service user perceptions, will be a key step in strengthening its validity.”

Corresponding adjustments are made under implications in the abstract:

“Further studies are needed to confirm the instrument’s sensitivity across diverse educational, professional, and cultural groups, and to evaluate its criterion validity using independent performance-based measures of competence.”

Also, the conclusion at the end of the Discussion section has been adjusted accordingly, now with this sentence included:

“Future validation studies should test the instrument against external performance-based measures and users’ perceptions.”

2 - *Clarify the purpose of the additional EFA analysis and its relevance to the research question.*
[This point is brought up by Reviewer 1: “I think also it would be useful to have more explanation of why the additional EFA analysis was conducted and what element of the research question this answered.”]

Our response: Here, we must apologize and express our gratitude for having this pointed out. We realize that the reference to EFE is actually unnecessary in the context of this article, and stems from an earlier draft. The factor analyses that provide the basis for such an approach were part of the original validation study. The relevant passage under *Data analyses* has now been revised to read as follows:

“We then applied multiple regression analyses to examine the influence of respondents' background characteristics on learning outcomes, both for the total score and for each of the domains. For all these dependent variables, learning outcome was calculated as the difference between pre- and post-test scores (post-test minus pre-test). The original validation study of the instrument (25) established a robust factor structure, supporting the assumption that domain scores reflect distinct but related constructs, thus providing a valid foundation for the regression analyses. All analyses were conducted using IBM SPSS Statistics, version 29 (SPSS Inc.).”

3 - *Acknowledge that students with the lowest pre-intervention scores showed the most improvement, which aligns with expectations but requires further explanation of the processes involved.*
[This point is brought up by Reviewer 1: “It is not surprising that those with the lowest scores pre intervention improved the most and processes such as ‘regression to the mean’ should be considered as an alternative explanation.”]

Our response: Thank you for this reminder, we agree that this point need some deepening regarding processes involved. Regression to the mean cannot be ruled out? as a possible process involved, though we argue it’s not a main explanatory factor as we see increased scores across all participants, not just those in the lower extremes. A revised section addressing these considerations is now included in the Discussion, and reads:

“It makes intuitive sense that prior trauma training level emerged as the main predictor of learning outcome, with the greatest pre-post improvement seen among participants with lower levels of prior training, as this group can be expected to possess the greatest potential for learning. Some regression to the mean may have been at play here (10), although the general increase in scores across all participants – not just those at the lower extremes – suggests it is unlikely to be the main explanatory factor. That lower prior training outweighed other predictors first and foremost points to TANDEM’s ability to detect learning outcomes regardless of participants’ type of workplace, target age group, role, or level of seniority.”

4 - *Explore why no significant change was observed in the Readiness domain. A deeper analysis of specific items or influencing factors (e.g., attitudes, personal readiness) is recommended.*

[This point is brought up by Reviewer 2: “The study mentions that no significant change was observed in the Readiness domain. It would be helpful to explore why this is the case. Perhaps a deeper analysis of the specific items within this domain could shed light on whether it was less responsive to the course or if other factors (e.g., prior attitudes, personal readiness) played a role.”]

Our response: Thank you, this comment really helps us to develop the article on an important point. A challenge with the Readiness domain is participants’ very high baseline scores, leaving minimal rom for variance. This may explain the lack of significant change but is – as your comment reminds us – none the less a concern. The limited variance reduces the variable’s value both as a practical guide and as a predictor in research. An important further development of the instrument would be to refine existing items or add new ones that capture more nuances of readiness, where your suggestion to delve deeper int personal attitudes, individual motivation (se point 6 below as well) and related aspects represents a natural direction. Against this background, we have developed the description of this issue, which now reads:

“Against this background, the lack of significant change in the Readiness domain warrants attention. This finding is, however, largely explained by the group's already high level of readiness at baseline, with a pre-survey total domain score of 15.5 out of 18, leaving limited room for further improvement. While this offers a plausible explanation, the limited variance within the scale remains a concern. When it comes to familiarity with the trauma field, the respondents in this study must be considered representative of the groups TANDEM is intended to serve, including in terms of their readiness. For the domain to retain any practical value, it may be necessary to revise existing items or introduce additional ones that capture a broader range of readiness-related aspects – preferably items that tap more directly into participants’ individual motivation and personal attitudes.”

Since a deeper exploration of participants’ individual motivation and personal attitudes was suggested also in comment 6, below, please confer our response to that point as well.

Adjustments made under implications in the abstract (se above), concerns this point as well:

“Future developments should test TANDEM across diverse educational, professional, and cultural groups, increase variance in the Readiness domain, and validate it against independent, performance-based competence measures.”

The point is also covered by the already mentioned adjustment in the conclusion of the Discussion section, now reading:

“Readiness domain should be further developed to allow for greater variance, and an additional measurement point should be introduced to assess the durability of the observed learning outcomes.”

5 - *Include participants from various educational and professional backgrounds to validate TANDEM's applicability across broader contexts and enhance generalizability.*

[This point is brought up by Reviewer 2: “While the study sample comes from Oslo Metropolitan University, it would be beneficial to include a more diverse group of participants from different educational backgrounds and professional sectors, as the authors mention. This could help validate TANDEM’s applicability across a broader range of contexts and enhance its generalizability.”]

Our response: Yes, this is an important point of caution when interpreting the results. As the reviewer notes, we have already highlighted the need to test TANDEM in samples with different educational and educational backgrounds. In response to the reviewer’s reminder that also cultural diversity should be sought (see point 8 below), and even variation when it comes to individual motivation and personal attitudes (see point 4 above and point 6 below). We have expanded our discussion of this limitation and included it under the new heading “Limitations and Next Steps” in the Discussion section, where the three first paragraphs now read:

“Despite the promising results, further studies are needed before any firm conclusions can be drawn about TANDEM’s overall sensitivity and applicability. One limitation of the study is that the sample consists solely of students from Oslo Metropolitan University, with, additionally, an overrepresentation of participants with three to four years of vocational education working primarily with adults in the healthcare sector. This distribution reflects the dominance of nursing students in the programs offered by the Faculty of Health Sciences (approximately 70%, see above).

A natural and important next step would be to include a more diverse group of participants from a broader range of educational and professional backgrounds. Preferably, this should include professionals outside the health sector, as well as individuals without formal professional training. As part of such a step, ensuring variation in regional and cultural representation should also be a priority, given Norway’s multicultural composition across both geographical regions and service structures.

Further testing of the instrument across professional and cultural contexts should also secure greater variation regarding participants' predisposition and attitude, where the current sample has a potential selection bias. Although participation in the course was mandatory, students had willingly enrolled in the broader educational program, suggesting a pre-existing interest or openness to the subject matter. This is demonstrated in the high Readiness scores at the outset, as commented on above, revealing a positive attitude toward adopting a trauma focus. Such attitude may also have influenced scores in other domains. Consequently, future studies should explore how TANDEM performs in groups that may approach the topic with more skepticism or hesitation.”

The comment is also covered by the already mentioned adjustments under implications in the abstract:

“Future developments should test TANDEM across diverse educational, professional, and cultural groups, increase variance in the Readiness domain, and validate it against independent, performance-based competence measures.”

Also, the beforementioned revised conclusion at the end of the Discussion, concern this point as well. It reads:

“Future validation studies should test the instrument against external performance-based measures and users’ perceptions. In these studies, samples should include participants from diverse educational, professional, and cultural backgrounds.”

6 - *Investigate factors such as individual motivation or personal trauma experiences that may influence learning outcomes but were not addressed in this study.*

[This point is brought up by Reviewer 2: “It would be useful to explore additional variables, such as the role of individual motivation or personal experience with trauma, which may also affect learning outcomes but were not fully addressed in this study. Including these factors could help refine the instrument’s sensitivity.”]

Our response: Yes, we also believe that personal factors related to participants' attitudes and experiences may influence their scores, and that this would be an interesting area to explore further. When it comes to personal experiences of trauma, it would be challenging within our Norwegian context to obtain permit to collect such sensitive information as part of a low-threshold screening for general use, such as TANDEM. However, this comment should also be viewed in light of the need to further develop the Readiness domain, addressed above. Including items that capture personal attitudes, expectations, and individual motivation, in order to obtain greater variance, would here represent a natural progression. We therefore refer you also to our response under point 4 above. The sequence included in the Discussion section addresses this point as well, with this sentence included:

“For the domain to retain any practical value, it may be necessary to revise existing items or introduce additional ones that capture a broader range of readiness-related aspects – preferably items that tap more directly into participants’ individual motivation and personal attitudes.”

7 - *Assess long-term retention of knowledge and skills acquired during the course to provide a more comprehensive evaluation of TANDEM's effectiveness.*

[This point was brought up by Reviewer 2: “The study primarily focuses on immediate pre-post changes. Future studies could explore the long-term retention of the knowledge and skills acquired during the course. This would provide a more complete picture of TANDEM's effectiveness in supporting sustained competence in trauma-informed care.”]

Our response: We agree and have now developed and expanded our description of this limitation and its impaction in the Discussion section. The sequence now reads:

“There are also uncertainties related to the extent to which TANDEM serves as a reliable measure of actual competence. One limitation is the timing of the data collection, occurring immediately after completion when the material was still fresh in participants’ minds. It remains uncertain how well the reported competence is retained over time. A logical next step would be to include a follow-up measurement point, for instance after six months. This has already been planned and will be integrated into the portal hosting the instrument.”

8 - *Conduct further validation studies in different regions or cultural contexts to confirm TANDEM’s reliability and sensitivity in diverse settings.*

[This point is brought up by reviewer 2: “While the study suggests promising psychometric qualities for TANDEM, further validation studies in different regions or cultural contexts would be beneficial to confirm its reliability and sensitivity across diverse settings.”]

Our response: Yes, we acknowledge the limitation of the narrow current sample, and the need for validations studies across cultural context. Under point 5, above, we have explained how we have solved this issue.
