# Supplementary material for: The sensitivity of TANDEM – A new measure of trauma competence
Source: PLoS One. 2026 Jan 28;21(1):e0339858. doi: 10.1371/journal.pone.0339858 (PMC12851466; doi:10.1371/journal.pone.0339858)
Supplement: Attachment — Submitted filename: Response to reviewers (2nd rev).docx [file pone.0339858.s005.docx]

**Dear editor**

Thank you for your latest constructive and clarifying comments on our paper, “The Sensitivity of TANDEM.” We have now accepted the tracked changes in the previous version of the manuscript so that the changes made in this round are clearly visible in the uploaded document titled “Revised manuscript with track changes (2nd rev)”. Below is an overview of the revisions we have made in response to the comments. In addition to these changes, the two references you suggested are now included, and the text references and reference list updated accordingly.

1. Clarify Cohort Size and Response Rate

“Recalculate and report the response rate using that exact denominator to ensure that the percentage aligns with 167 respondents.”

“I had not previously noticed that the first paragraph under ‘2.2 Sample’ says that each year an average of 90 students complete this course so over 3 years this would be 270. The response rate of 167 is reported as 74% of the cohort but 167 is 61.9% of 270. I would give the exact number of people who completed the course over the 3 years rather than the average per year.”

Our response: Thank you – this really highlights the need to be more exact and precise. Our dilemma is that (to the best of our ability to track back) only around three-quarters of the total number of students actually attended the course lectures. Therefore, including the full cohort in the sample would result in a response rate that is somewhat “unfairly”. However, based on your reminder, we believe that this is still the correct and most reliable way to approach it, so we have now chosen to base the calculation on the exact number of students who completed the course in each of the three years. The first paragraph under the heading “Sample”, which presents these figures, now reads as follows:

“The final sample consisted of a total of 163 respondents. These were from a total cohort of 339 students who completed the course on Mental Health, Trauma and Crisis in a Life Course Perspective, at OsloMet, January 2021 (121 students), January 2022 (110 students), and January 2023 (108 students), respectively. Of these, 167 (49,3%) (n2021=61; n2022=53; n2023=49) submitted responses on both the pre- and the post TANDEM surveys. Four responses from the 2022 cohort had mostly empty fields either in the pre- or post-survey and were subsequently excluded from the sample. As a result, the final total response rate was adjusted to 48,1% (N=163). However, TANDEM was administered in connection with lectures which, to the best of our estimate, were attended by only three-quarters of the students. Therefore, among those who actually followed the course, the response rate would be higher.”

2. Align Domain Count with Nordanger et al. (Ref. 25)

“The original validation by Nordanger et al. employed nine domain scores (six main domains, three split into subdomains). Please clarify whether your use of six domains reflects aggregation of those subdomains, or else adjust your description so that the number of domains matches the validation reference. Ensure consistency between the manuscript text, your tables/figures, and the factor‐analysis results reported in Ref. 25.”

«The section on multiple regression analysis in the ‘2.5 Statistical analyses’ section now states the ’validation study of the instrument (25) established a robust factor structure, supporting the assumption that domain scores reflect distinct but related constructs, providing a valid foundation for the regression analyses’. However, the ref 25 Nordanger et al paper uses 9 domains in their validation analyses (tables 1-5). These are the 6 main domains but 3 are split into two subdomains and all 9 were included separately in their factor analysis as far as I can tell. I don’t think this is a big issue, but the number of domains should be consistent with the reference.”

Our response: We are truly grateful for such a careful and conscientious reading – we recognize that, as it stands, this issue is not presented in a sufficiently clear and transparent manner (though we are glad that the reviewer does not regard it as a big issue, provided it is clarified). Regarding the names of the domains, the domain referred to as "Practices" in the initial validation study (ref. 27) has actually later been formally renamed to "Skills" by the development group. The change was needed because the term "Skills" better represents the domain's items, and also because "practice" is often used more broadly in similar contexts (e.g. trauma-informed practice). In the current paper we therefore need to retain the term "Skills", while also informing the reader about the change from the first study.

However, in the current study, we have also made an unnecessary and misleading name change from "Working Culture" in the original study to "Organisational Culture" in this one. On this point, we will revert to the original term. Cleaning up and ensuring consistency when it comes to the count and names of domains involves some revision in five places throughout the document:

Firstly, where the TANDEM domains are introduced for the first time – in the second paragraph under heading 1.1 – it now states:

TANDEM includes 55 test items spread across six main domains: Readiness, Agency, Reflexivity, Knowledge, Skills, and Work Culture. The Knowledge, Skills, and Work Culture domains each contain two subdomains (see Method, below), bringing the total to nine domains. Note that the Skills domain was named “Practice” in the first validation study (27) but was renamed at a later stage.

Secondly, also under 1.1., second last paragraph page 4, the sentence describing the scale reliability and model fit of domains now reads as follows:

“All domains and subdomains showed good scale reliability, and each also corresponded with a one-dimensional model.”

Thirdly, where the measures for the current study are introduced in the first paragraph under heading 2.1 in the Method section, it now reads:

All 55 test items in TANDEM are scored on a six-point ordinal scale, typically ranging from ‘To a small degree’ (1) to ‘To a great degree’ (6). The instrument is presented in its entirety in the article by Nordanger and colleagues cited above (27). For the purpose of the current study, the analyses primarily deal with the main domains, meaning that the subdomains within Knowledge, Skills, and Work Culture are treated collectively. The distribution of test items across the domains is as follows:

Fourthly, under the same 2.1. heading, point 5, where the items of the Skills domain are presented, we now have added the following suffix:

“As noted above, this domain was named “Practice” in the original validation study (27).”

Fifthly, the last domain, named “Organisational culture” in the previous version, has been renamed to “Work culture” throughout the manuscript.

3. Specify Formative vs. Reflective Measurement Model

" Explicitly state whether TANDEM is conceptualized as a reflective model (single latent construct driving domain scores) or a formative model (competence formed by distinct domain scores)"

"I think it would be good to be explicit about whether this was based on a formative or a reflective measurement model. That is theoretically, for a reflective model there is a single underlying latent construct of trauma competence that drives all the scores on this measure. Alternatively in a reflective model trauma competence is made up of, or formed by, competence in the different areas/domains covered by the measure. This entails that scores on the domains will not necessarily be highly correlated and therefore they can be regressed separately. I think it would be helpful to readers to be more explicit about the formative versus reflective nature of the measure under discussion."

Our response: We fully agree that whether the instrument is based on a reflective or formative model is an important piece of information for any reader. The development of TANDEM, as well as the analytical approach used in the initial validation study, was based on a reflective model. This is now explicitly stated in the paragraph under 1.1. in the introduction where the instrument and the initial validation study results are presented – i.e. the second last paragraph page 4, which is now expanded with the following two sentences:

“TANDEM is conceptualised as a reflective model (7), based on the notion that the instrument as a whole captures an underlying construct of trauma competence, while the items within each domain capture specific aspects of this competence. This understanding also formed the basis for the initial validity analyses (27).”

4. Address Regression‐to‐the‐Mean Concern

"In your Results or Discussion section, briefly acknowledge the mathematical coupling between baseline scores and change scores (e.g. Clifton & Clifton, 2019, Trials 20:43), and explain how your analytical approach mitigates any undue bias from regression to the mean."

"My comment about the regression to the mean referred to the correlation between baseline score and the learning outcome ‘Learning outcome was most strongly correlated with low baseline score (r = -.68, p < .001)’. This is because mathematically it can be shown that there is always a correlation between the baseline score and the change score. This is shown by equation 4 in this paper Clifton, L., Clifton, D.A. The correlation between baseline score and post-intervention score, and its implications for statistical analysis. Trials 20, 43 (2019). <https://doi.org/10.1186/s13063-018-3108-3>"

Our response: Yes, we now understand more clearly the nature of the concern and what the reviewer is requesting at this point. Although we continue to believe that the absence of a regression to the mean pattern among participants with higher pre-scores is a positive indication regarding the influence of this effect, we recognise that it nonetheless must be acknowledged as a possible explanatory factor for the observed correlation between lower baseline scores and learning outcome. Since our analytical approach cannot say to fully control for this effect, we think that the most appropriate and transparent way to address it is as a point of caution or limitation. Accordingly, this paragraph in the Discussion section addressing this issue (last on page 13) now reads as follows:

“It makes intuitive sense that the level of prior trauma training emerged as the main predictor of learning outcome, with the greatest pre–post improvement observed among participants with lower levels of prior training, as this group would be expected to have the greatest potential for learning. This finding must, however, be interpreted with some caution, as it has been mathematically demonstrated that – as part of the statistical phenomenon known as “regression to the mean” (8) – there will always be a correlation between baseline scores and change scores. That said, the general increase in scores across all participants – not just those at the lower end of the scale – suggests that regression to the mean is not the sole explanatory factor. It is still reasonable to interpret the finding as an indication of TANDEM’s ability to detect learning outcomes regardless of participants’ workplace type, target age group, role, or level of seniority. In this sense, TANDEM shows promise in handling the diverse contexts of services, target groups, and TCP formats that competence centres and higher education institutions engage with, and thus fulfilling its intended purpose.”
