# Supplementary material for: The sensitivity of TANDEM – A new measure of trauma competence
Source: PLoS One. 2026 Jan 28;21(1):e0339858. doi: 10.1371/journal.pone.0339858 (PMC12851466; doi:10.1371/journal.pone.0339858)
Supplement: Attachment — Submitted filename: Respons to reviewers (3rd revision).docx [file pone.0339858.s006.docx]

**General comments:**

All changes from the earlier review rounds, which were well received by the reviewer, have been retained and are carefully preserved in the new manuscript.

The revisions made to address Reviewer 3’s requests involved substantial modifications, including additional analyses and a completely new presentation of results. For this reason, the resubmitted manuscript is uploaded as a new document without tracked changes.

Some sections have been moderately condensed to accommodate space requirements resulting from the expansion of other sections; nevertheless, again, all responses to previous feedback remain fully preserved.

As previously clarified, the dataset now includes an additional cohort of students who completed their program prior to this latest review round.

The extensive work required for the new analyses also entailed a shift in workload and responsibilities, with Rolf Gjestad, who had primary responsibility for the statistical analyses, now listed as first author. This change was communicated to you in advance.

The abstract is updated to represent the revised version.

**INTRODUCTION – reviewer’s recommendations for improvements:**

*1. The two sub-questions are descriptive rather than hypothesis-driven. Re-casting them as formal, directional hypotheses would sharpen the study’s focus.*
 ***Response:*** Thank you, we fully agree. This paragraph is now rewritten and reads: “As a collaboration between RVTS West and SHA at OsloMet, this study investigates whether TANDEM can detect changes in learning outcomes from trauma TCPs, using data from four student cohorts who completed the survey in the TCP on Mental Health, Trauma, and Crisis in a Life Course Perspective. This overarching research question is specified into two directional hypotheses:

1. Participants’ TANDEM scores are expected to increase from pre- to post-TCP, reflecting measurable improvements in trauma competence.
2. Participants’ characteristics will predict level and change TANDEM scores, with greater improvements expected among those with less prior training.”

*2. Empirical evidence (e.g., pilot data) showing why existing tools under-perform in Norway would strengthen the justification for a new instrument.*
 ***Response:*** Thank you for reminding us to make this clearer. ARTIC, which was considered the most relevant instrument for translation, has not yet been tested in Norway, so the following has now been added here: “There have been no previous attempts to apply ARTIC in a Norwegian context, and based on a careful review, it was evaluated that a translated version would not serve the intended purpose. The scale was considered too basic for the competence level within Norwegian services, and it was evident that it was developed for contexts that do not reflect the total ban on corporal punishment mandated by Norwegian legislation (27).”

*3. Consistent terminology—decide on “course”, “programme”, or “TCP” and use it uniformly.*
 ***Response:*** Thank you for pointing this out. You are right, there was some inconsistency in our use of terminology, as “programme” was used both for the specific course at OsloMet and for TCP programmes in general. This has now been made consistent: course is used only for the specific OsloMet training, while TCP is consequently used when referring to trauma competence programmes in general.

*4. Cite current psychometric guidance (e.g., COSMIN responsiveness standards) to ground the work in best-practice measurement science.*
 ***Response:*** Thank you for directing our attention to the COSMIN guidelines. We have now included references to these guidelines and revised the text in several places to clarify how the study adheres to them. For example, on page 5 we now state: “Adhering to best-practice measurement science and the COSMIN reporting guidelines (34), a critical aspect to examine is the instrument’s responsiveness, or sensitivity to change (31–33), defined here as its ability to detect meaningful changes (31, 34) in respondents’ scores from before to after participating in a TCP.”

**METHODOLOGY – reviewer’s recommendations for improvements:**

*1. The single-arm design limits causal inference. Explain why a comparison group was not feasible and temper causal language.*
***Response:*** Thank you for giving us the opportunity to clarify this. We fully agree that including a comparison group, ideally through an RCT design, could strengthen causal inference regarding the learning effect. However, the present study was designed primarily to evaluate the measurement of learning outcomes rather than to test causal effects of the training intervention itself. For this reason, we did not consider an RCT or other comparison group design to be appropriate in this context. Likewise, we see no feasible way to implement a non-randomized control group, or to identify the control group that would appropriate for this kind of study.

*2. No attrition analysis is provided; a participant flow diagram would reveal potential bias.*
***Response:*** Thank you. We have now included a much larger sample and also used data from subjects with responses from one measurement occasion only, as we now use full information maximum likelihood estimation (FIML) as the estimator for measurement invariance CFA and for the unconditional and conditional latent growth curve (LGC) models. Thus, all available data is used for data analysis under the “missing at random” assumption (MAR). This strategy represents an improvement compared to the more standard analyses like ANOVA / t-test with repeated measures that assumes missing completely at random (MCAR). We now have to assume missing at random, which is a less restrictive assumption. This makes the analyzed sample size easy to report, as is now done in the Sample section (2.2). Due to this, we do not see it necessary to use a flow diagram.

*3. Scoring excludes respondents with any missing items, which may distort change estimates.*
***Response:*** Yes, thank you for this comment. We agree. As described above, we now use all available data under the MAR assumption with the FIML estimator in Mplus.

*4. Consider multiple imputation or prorating.*
***Response:*** Yes, we did consider the use of multiple imputation (MI). However, we chose to use Full Information Maximum Likelihood (FIML), which has been shown to often produce estimates comparable to those obtained through MI under the assumption of data missing at random (MAR). Even if the potential inclusion of auxiliary variables in FIML and predictors in MI, which possibly may be more in numbers, these two strategies may give some differences. However, given the structure and amount of our data, FIML was deemed appropriate and efficient for handling missing responses.

*5. Effect-size metrics (Cohen’s d, SRM) and confidence intervals are mandatory for interpreting practical significance yet are absent.*
***Response:*** Thank you, both effect sizes and confidence intervals for the change scores are now included.

*6. Dichotomising continuous predictors (e.g., workplace type) in regression analyses discards information and inflates error rates; retain original scales or use dummy coding.*
***Response:*** Thank you. Yes, we are aware of this issue. The strategy for handling these variables is clarified in the revised final paragraph of the “Statistical Analysis” section (2.5), where we describe the rationale and focus of the predictor variables.

*7. The EFA results are densely embedded in text; a supplementary table with factor loadings and explained variance would improve clarity.*
***Response:*** Thank you. We used Confirmatory Factor Analysis (CFA), and the standardized factor loadings are now presented in Supplementary Table 2. To simplify the presentation, we have included the factor loadings from the unconstrained models with freely estimated parameters, not factor loadings for all invariance models. This approach is also described in the manuscript.

*8. A multigroup CFA to test pre/post measurement invariance would confirm that score changes reflect true change rather than shifting factor structure.*
***Response:*** Thank you for this comment, which prompted us to thoroughly revise our theoretical perspective regarding the measurement models, conduct and report measurement invariance analyses, and reflect more deeply on the interpretation of the results. Following substantial revisions, the manuscript now clarifies that we distinguish between reflective and formative dimensions, acknowledging the hybrid nature of the instrument. Only reflective measurement models require correlated measurement indicators as caused by the underlying constructs, which makes reliability analyses (e.g., Cronbach’s alpha, McDonald’s Omega) and factor analyses appropriate. In contrast, formative constructs do not require inter-item correlations, as the indicators represent distinct components causing the construct rather than manifestations of the construct as (a latent dimension / trait). After careful consideration, including a revisiting of the scale development process and the original intent behind the constructs, we now conceptualize Knowledge, Skills, and Work Culture as formative, and Readiness, Agency, and Reflexivity as reflective constructs. Consequently, the latter dimensions were analyzed using measurement invariance models, testing for configural, weak, strong, and strict invariance across time.

*9. Data-sharing plans do not yet meet journal policy—deposit de-identified data in a public or restricted-access repository.*
***Response:*** Data has now been stored in our institution’s repository, and will be available without restriction; we are awaiting the DOI nr. within a few days.

**RESULTS – reviewer’s recommendations for improvements:**

*1. Add paired-samples Cohen’s d (or SRM) and 95 % CIs to all change estimates.*
***Response:*** Yes, thank you. This is now included in the manuscript.

*2. Provide baseline distribution data to substantiate the proposed ceiling effect in the ‘Readiness’ domain.*
***Response:*** The standard deviation, both for the observed scores and the estimated intercept scores in the LGC model, is included in Table 3. We think that a ceiling effect is present as the mean score approaches the maximum possible value, and the standard deviation is low, indicating limited variability among responses. This suggests that participants may have reached the upper limit of what the instrument can measure, potentially constraining the ability to detect further improvement or differentiation.

*3. Report standardised β coefficients and CIs for regression predictors to show relative importance.*
***Response:*** Yes, thank you – please see the new, much more detailed Table 5, where beta weights are now included.

*4. Include the full factor-loading matrix and variance explained for pre- and post-EFA, ideally in an appendix.*
***Response:*** We have used CFA, not EFA. As described above, the factor loadings are now given in supplementary table 2.

*5. A simple forest plot or line graph illustrating mean change by domain would make the results more digestible.*
***Response:*** Thank you for the suggestion. With the more extensive presentation of results in Table 5 and relatively few statistically significant findings, we believe that a forest plot would not contribute meaningfully to further clarifying the results. The current tabular format allows for a more detailed and transparent presentation of both estimates and statistical significance.

**DISCUSSION – reviewer’s recommendations for improvements:**

*1. Address potential response-shift bias explicitly - future studies could use “then-retro” ratings or structural-equation methods.*
***Response:*** Thank you. Yes, the now included measurement invariance analyses on the reflective measurement dimensions within the SEM framework and discuss the possibilities for response-shifts in these. Thank you also for the comment about “then-retro” ratings. While we did not apply then-retro ratings in the current study, we acknowledge that this approach may be relevant in future research, particularly in educational settings where participants’ baseline understanding may be limited or subject to response shift bias. Given our focus on measurement and the use of longitudinal data with pre–post assessments, we prioritized evaluating measurement invariance to ensure valid comparisons over time. However, we agree that future studies could benefit from considering then-retro designs as a complementary strategy to capture perceived change more accurately. We have now included a sentence about this under “Limitations and next steps”.

*2. Compare observed effect sizes (once reported) with those from ARTIC or similar instruments to situate findings quantitatively.*
***Response:*** Thank you, this strengthens the scientific grounding of the study. We have now added the following to the second paragraph in the Discussion section: “The overall effect size detected by TANDEM (d = 1.37) appears to fall within a similar range to ARTIC, which may be the most relevant instrument for comparison (see Introduction). Among the few studies we identified that have examined ARTIC as a pre–post measure of trauma training outcomes, reported effect sizes range from approximately d = 0.5 among multi-disciplinary clinical staff (Gouveia et al., 2023) to d = 1.35 among school personnel (Kelly et al., 2021).”

*3. Temper concluding statements such as “the basis for using TANDEM is significantly strengthened” by noting design constraints.*
***Response:*** Yes, we agree. In addition to more balanced formulations throughout the Discussion, the concluding remark has now been phrased more modestly and reads as follows: “TANDEM shows promise as a tool for supporting the implementation, evaluation, and development of trauma competence programs in health and social care contexts. The instrument captured changes in scores from before to after the TPC in a way that appear meaningful against the background of the course curriculum. Future developments should test TANDEM across diverse educational, professional, and cultural contexts, increase variance in the Readiness domain, investigate interpretative changes in the Agency and Reflexivity domains, and validate the instrument against independent, performance-based competence measures.”

*4. Offer a clearer roadmap for next steps: (1) measurement-invariance testing; (2) longer-term follow-up; (3) broader professional samples; (4) objective outcome correlations.*
***Response:*** Yes, we hope you now consider this adequately addressed through the revised summary (above), as well as the new reflection added regarding the measurement model at the end of the Discussion (before the Limitations section).
