# Supplementary material for: The sensitivity of TANDEM – A new measure of trauma competence
Source: PLoS One. 2026 Jan 28;21(1):e0339858. doi: 10.1371/journal.pone.0339858 (PMC12851466; doi:10.1371/journal.pone.0339858)
Supplement: Attachment — Submitted filename: Response to reviewers (4th revision).docx [file pone.0339858.s008.docx]

**Response to reviewers, 4^th^ revision**

**Editor asked us to respond to each of the comments and requests below (stated as “What I’m recommending to the authors”):**

1. **Finalize the Sample section** by keeping only the updated paragraph with the 339 denominator, 167 paired responses, 4 exclusions, final N=163, and overall 48.1% response rate; remove the legacy “70%” language.

*Response: It appears that an error has occurred on our side. As described in the previous response, our intention was to upload a clean document without tracked changes, given the extent of the revisions. However, it seems that we inadvertently uploaded a file in which some remnants of the previous version were still present. That explanation is no longer relevant now that all respondents and all missing data have been included. The correct version, as it now stands, reads:* “The sample was drawn from a total cohort of 500 students who completed the course on Mental Health, Trauma and Crisis in a Life Course Perspective, at OsloMet, January 2021 (121 students), January 2022 (110 students), January 2023 (108 students) and January 2024 (161 students), respectively. Of these, 370 students (n2021=97; n2022=98; n2023=94; n2024=81) submitted responses on the TANDEM surveys, representing a response rate of 74%.”

2. **Clarify invariance testing** by stating the exact decision rules (e.g., ΔCFI ≤ .01; ΔRMSEA ≤ .015; non‑significant χ² difference), and correct the “reduction in χ²” phrasing if reversed. Remove or clearly sequester the residual **EFA** paragraph.

*Response: Thank you. We are still learning from this process. We have now revised the relevant section and expanded the strategy described in the Statistics section. As you suggested, we have updated the supplementary table on measurement invariance and included model fit indices for all steps in the constraint testing procedure; Chi-square, CFI, TLI, and RMSEA, along with the absolute differences in these indices across steps.*

*The EFA paragraph was not intended for inclusion in the final version of the manuscript and appears to have been another remnant from an earlier draft due to the upload error mentioned above. We apologize for this oversight, and we have now ensured that it has been removed entirely.*

3. **Make the analysis outputs visible in**‑**paper.** Ensure pre–post effect sizes **with 95% CIs** and clearly labeled **standardized vs unstandardized** coefficients appear in the main tables, not only in DOCX supplements. Briefly acknowledge multiplicity and whether any FDR‑style control was considered.

*Response: Effect size (ES) with 95% CI is now included in table 3. Unstandardized and standardized regression coefficients are reported in table 5. In addition, standardized beta-weights are added to the text in the 3.4 “Predictors of learning outcomes” paragraph, for results not given in the table. The last version included unstandardized results with p-values, only. Regarding multiplicity (type-I error, familywise error rate (FWER), and false discovery rate (FDR), we have now added a paragraph about statistical testing and this topic at the end of the statistical analyses section (2.5).*

4. **Justify or reduce dichotomization** of Target age group, Workplace type, and Role type; if feasible, rerun with dummy/ordered terms and report sensitivity analyses.

*Response: Thank you. The rationale for this is now explained more clearly. We have also moved this section so that the explanation now follows directly after the description of the relevant variables and their response categories under “2.1. Measures”. This makes the reason for recoding more immediately apparent to the reader.*

5. **Harmonize terminology.** Pick either “Work culture” or “Organisational/Organizational culture” and use it consistently across text and tables; choose UK or US spelling and apply uniformly.

*Response: Thank you. It should now be referred to consistently and exclusively as work culture throughout the document, including in the tables, and spelling should now be consistently US.*

6. **Lock down data availability** by inserting the repository DOI/URL and adding a short codebook (variables, scales, and recodes).

*Response: This information is now reflected in the PLOS ONE Editorial Manager system. The full data matrix is available at [https://doi.org/10.18712/NSD-NSD3333-V1](https://doi.org/10.18712/NSD-NSD3333-V1) in the Sikt repository (Sikt – Norwegian Agency for Shared Services in Education and Research), and the DOI will be activated upon publication of the article. The codebook has been uploaded as Supporting Information in Editorial Manager and will also be available in the same Sikt repository.*

7. **Align authorship metadata** so the author order and corresponding author in EM match the manuscript.

*Response: The author order has been updated in the submission metadata in PLOS ONE Editorial Manager system, so that it now matches the order in the manuscript, with Rolf Gjestad listed as first author.*

8. **Tidy presentation** by removing all placeholders/duplications and ensuring the final table/figure set conforms to PLOS formatting.

*Response: Placeholders and duplications have now been removed, and all tables have been inserted into the manuscript in accordance with PLOS ONE formatting standards. However, we were not able to organize Table 5 (and Supplementary Table 1) in a way that avoids the need for a landscape page in the manuscript. We hope this will be acceptable, and that these tables can be rotated during the production process if needed.

Note that the two supplementary tables (S1_Table and S2_Table) have been uploaded as Supporting Information in the PLOS ONE Editorial Manager system.*
